# The association between disability progression, relapses, and treatment in early relapse onset MS: an observational, multi-centre, longitudinal cohort study

**DOI:** 10.1038/s41598-023-38415-z

**Published:** 2023-07-18

**Authors:** Valery Fuh-Ngwa, Jac C. Charlesworth, Yuan Zhou, Ingrid van der Mei, Phillip E. Melton, Simon A. Broadley, Anne-Louise Ponsonby, Steve Simpson-Yap, Jeannette Lechner-Scott, Bruce V. Taylor

**Affiliations:** 1 Present Address: Menzies Institute for Medical Research, University of Tasmania, 17 Liverpool St, Hobart, TAS 7000 Australia; 2 Present Address: Menzies Health Institute Queensland and School of Medicine, Griffith University, Gold Coast, QLD 4222 Australia; 3 Present Address: Florey Institute for Neuroscience and Mental Health, Parkville, VIC 3052 Australia; 4 Present Address: Neuroepidemiology Unit, Center for Epidemiology and Biostatistics, The University of Melbourne School of Population & Global Health, Melbourne, VIC 3053 Australia; 5 School of Medicine and Public Health New Lambton, Hunter New England Health, New Lambton Heights, NSW Australia; 6 Department of Neurology, The University of Newcastle Hunter Medical Research Institute, New Lambton Heights, NSW Australia

**Keywords:** Multiple sclerosis, Epidemiology, Disease-free survival

## Abstract

The indirect contribution of multiple sclerosis (MS) relapses to disability worsening outcomes, and vice-versa, remains unclear. Disease modifying therapies (DMTs) are potential modulators of this association. Understanding how these endo-phenotypes interact may provide insights into disease pathogenesis and treatment practice in relapse-onset MS (ROMS). Utilising a unique, prospectively collected clinical data from a longitudinal cohort of 279 first demyelinating event cases followed for up to 15 years post-onset, we examined indirect associations between relapses and treatment and the risk of disability worsening, and vice-versa. Indirect association parameters were estimated using joint models for longitudinal and survival data. Early relapses within 2.5 years of MS onset predicted early disability worsening outcomes (HR = 3.45, C.I 2.29–3.61) per relapse, but did not contribute to long-term disability worsening thereinafter (HR = 0.21, C.I 0.15–0.28). Conversely, disability worsening outcomes significantly contributed to relapse risk each year (HR = 2.96, C.I 2.91–3.02), and persisted over time (HR = 3.34, C.I 2.90–3.86), regardless of DMT treatments. The duration of DMTs significantly reduced the hazards of relapses (1st-line DMTs: HR = 0.68, C.I 0.58–0.79; 3rd-line DMTs: HR = 0.37, C.I 0.32–0.44) and disability worsening events (*1st-line DMTs:* HR = 0.74, C.I 0.69–0.79; 3rd-line DMTs: HR = 0.90, C.I 0.85–0.95), respectively. Results from time-dynamic survival probabilities further revealed individuals having higher risk of future relapses and disability worsening outcomes, respectively. The study provided evidence that in ROMS, relapses accrued within 2.5 years of MS onset are strong indicators of disability worsening outcomes, but late relapses accrued 2.5 years post onset are not overt risk factors for further disability worsening. In contrast, disability worsening outcomes are strong positive predictors of current and subsequent relapse risk. Long-term DMT use and older age strongly influence the individual outcomes and their associations.

## Introduction

Multiple sclerosis (MS) is clinically a complex disease with two seemingly disparate clinical phenotypes namely: relapses and inexorable disability progression. These two features can occur concurrently or be temporally separated and are the basis for classification of MS into the well-recognised clinical phenotypes of relapsing-onset MS (ROMS) and progressive onset MS (POMS). However, despite this dichotomy, the contribution of relapses to disability worsening, and vice-versa, is not well understood. Understanding the interactions between these 2 clinical phenotypes; and the temporal interaction with each other and treatment may provide insights into the underlying mechanisms of disease progression and consequently the treatment of MS.

Previous studies have demonstrated the direct predictive values of early relapses on disability worsening outcomes in the short^1,2^, and long term^3–10^; whereas others^11–15^ have reported a dissociating and decreasing impact, of either early or late relapses on longer term disability accrual. However, there is considerable evidence for there being no direct effect of relapses on long-term disability accrual from observational studies in ROMS^1,12,14–21^ and secondary progressive (SPMS)^14,22–24^. For instance, in Tremlett et al.^11,25^ disability accrual in patients with SPMS was attributed to the effect of chronologic age and disease duration, with the milestones of EDSS (Expanded Disability Status Scale) 4 and 6 being reached on predefined visits not overtly influenced by relapses. These finding has been recently confirmed in ROMS^15^. In this regard, SPMS can be regarded as ROMS in which the relapsing phase has ended^5,26^. By reasoning, if the progressive accumulation of disability in SPMS or POMS occurs regardless of relapses, then it could be hypothesised that relapses may have little bearing on subsequent disability worsening outcomes in ROMS. But whether relapses have an independent effect on disability accrual during and after the relapsing phase of ROMS is unclear. Kappos et al.^18^ showed that most disability accumulation was not predicted by relapses; and Ahrweiller et al.^12^ demonstrated a direct decreasing impact of late relapses on disability worsening.

There is strong evidence from large phase 3 clinical trials that disease-modifying therapies^27,28^ (DMTs) (and potentially use of vitamin D_3_ [VitD] supplements^29–31^) approved for the treatment of relapses modulate a variety of largely inflammatory molecular pathways to reduce relapse rates, and the accumulation of disease burden, particularly, as measured by new MRI T2 white matter lesions (T2L)^20,29,32^. However, their effects on long-term disability accrual are less clear, although they are likely to have a positive long-term benefit^20,32^. Although the results of VitD supplementation trials in established MS have been underwhelming^29–31^, prior work has shown a synergistic effect of VitD and DMTs in modulating relapse risk^33–35^. However, how the use of DMTs interact to modulate the association time-dynamic associations between relapses and disability accrual is not fully understood.

Further, established genetic variants associated with MS risk^36^, particularly single nucleotide polymorphisms (SNPs) have been shown to have additional prognostic values^20,37–42^ in predicting MS relapses^20,37^ and the disability worsening^20,38,40–42^, and are vital instruments for investigating indirect associations in the presence of known confounders. Therefore, by jointly modeling the underlying correlations and the longitudinal processes governing the cumulative effects of these risk variants and the individual outcomes (relapses and disability worsening), a better understanding of the associations between these outcomes can be further elucidated. In this vein, we aimed to examine the indirect contributions of MS relapses to the risk of worsening of disability, and vice-versa; and whether the use of DMTs have the potential to modulate the time-dynamic association between relapses and disability worsening outcomes, as well as the individual associations.

## Materials and methods

### Data and study design

We used clinical and genetic data pooled from the multi-centre (Brisbane, Newcastle, Geelong and Western Victoria, and Tasmania) Australian Longitudinal Prospective Cohort Study (AUSLONG) of MS progression^43^. Between 2003 and 2006^43^, participants were recruited into the AUSLONG study following a first clinical diagnosis of a central nervous system demyelinating event. Initial data extracted included clinical and demographics quantitative variables: the age at onset, T2 white matter MRI lesion load, number of previous relapses (relapse counts), body mass index (BMI), functional systems scores; and categorical variables: sex, vitamin D_3_ supplement status, study site (latitude), MS subtype (ROMS or CIS), and expanded disability status scores (EDSS). T2 lesion loads were measured using the 2015 MAGNIMS consensus guidelines^44^. The AUSLONG study has ethical approval from the Tasmanian Health and Medical Research Ethics Committee (ref: H0010499, 01/-5/2009). All experiments (blood collection, genotyping, and clinical examinations) were conducted following strict guidelines. Written informed consent was obtained from all subjects and/or their legal guardian. SNP genotype data was available for 199 of the ~ 233 (~ 200 autosomal, and ~ 33 HLA) MS risk variants published by the International MS Genetics Consortium (IMSGC)^36^ Quality control of the genotype data was conducted using established protocols^45^, and described previously in Fuh-ngwa et al.^41^.

### Inclusion and exclusion criteria

**Figure 1:**
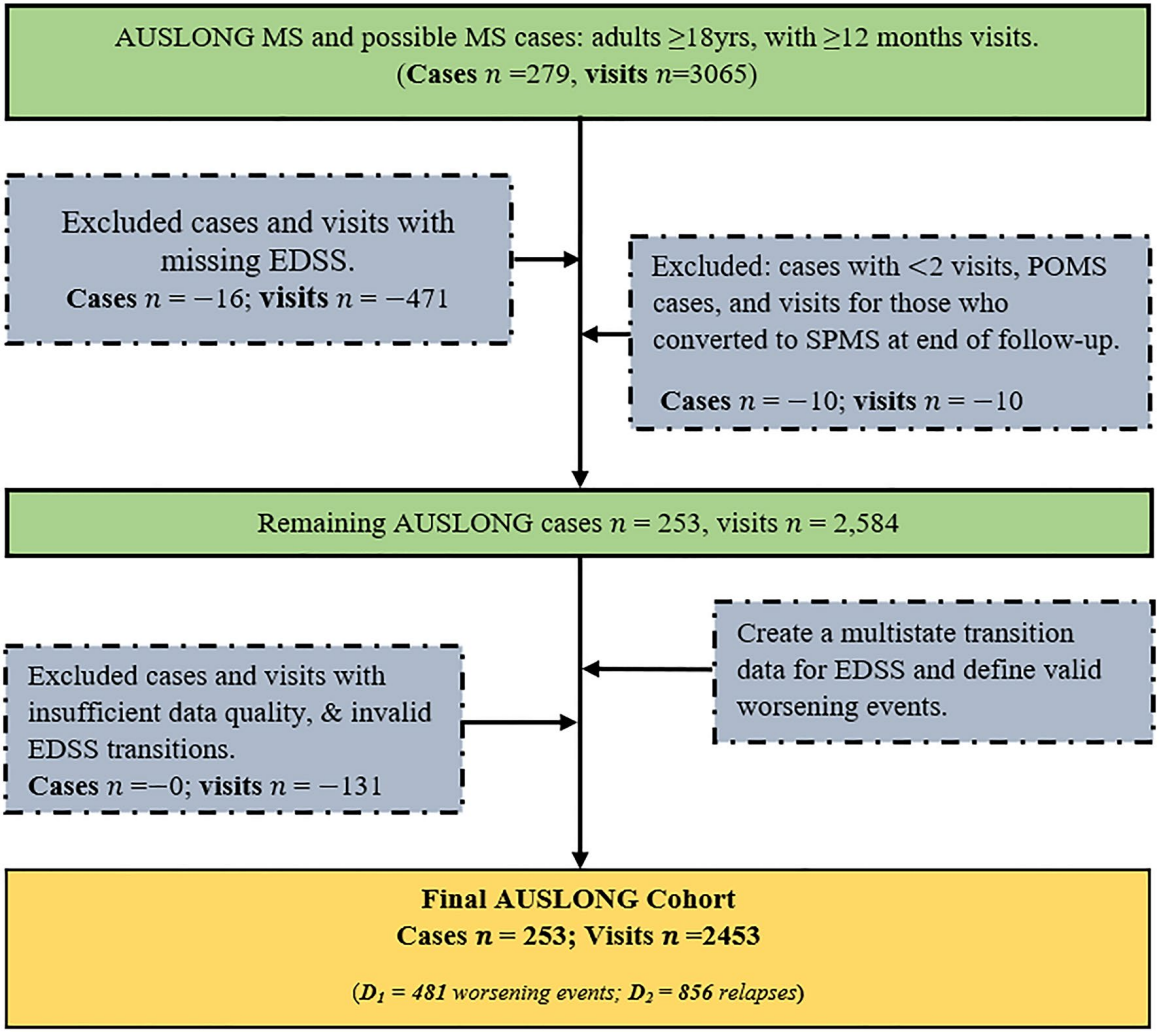
A flowchart of AUSLONG case data extraction and selection.

Figure 1 describes the inclusion criteria for the initial extraction of MS cases. MS cases were defined using the 2017 McDonald criteria^46^. Using these criteria (Fig. 1), we analysed 253 cases with up to 15 years of follow-up after onset and with 2453 measured EDSS transitions; and who had been diagnosed as either ROMS (n = 219) or remained as clinically isolated syndrome (CIS) by 15th year review (n = 34).

### Outcomes definitions

#### Confirmed MS relapses

MS relapses were defined as the appearance of new or worsening neurological symptoms or worsening of previously stable or improving pre-existing neurological deficits (not caused by fever or a known infection) and lasting more than 24 h. Confirmed MS relapses were defined as MS relapses accompanied by a clinically meaningful change in EDSS (e.g. at least 1-point increase in EDSS). Only relapses confirmed during EDSS visits were included in the statistical analysis.

#### Worsening of disability

Disability accumulation was defined based on EDSS*.* EDSS was measured by trained and certified neurologists, confirmed face-to-face initially at baseline, 2.5yrs, and 5yrs. We also included validated telephone EDSS^47–51^ that was collected yearly from 5yrs and up to 15 years post onset^52^. Disability worsening outcomes were based on EDSS, and were statistically defined using a first-order Markov’s assumption that preserves the continuous-time evolution of EDSS^53^. Specifically, we hypothesised that the current EDSS score depended on the previous score, and the EDSS transition time is continuous rather than discrete^53^. Additionally, valid EDSS transitions were obtained using a transformation function “*msm2Surv*” in the *mstate* R-package^54^. Additional definitions of “*worsening*” (at least 1 point increment in EDSS in) versus “*improved*” (at least 1 point decrease in EDSS) events was supported by the literature^1,55–58^. The observation time for EDSS was defined as the continuous time elapsed since onset or MS diagnosis until the current visit (see Appendix 1).

### Statistical analysis

#### Analysing the time-to-relapses

**Figure 2:**
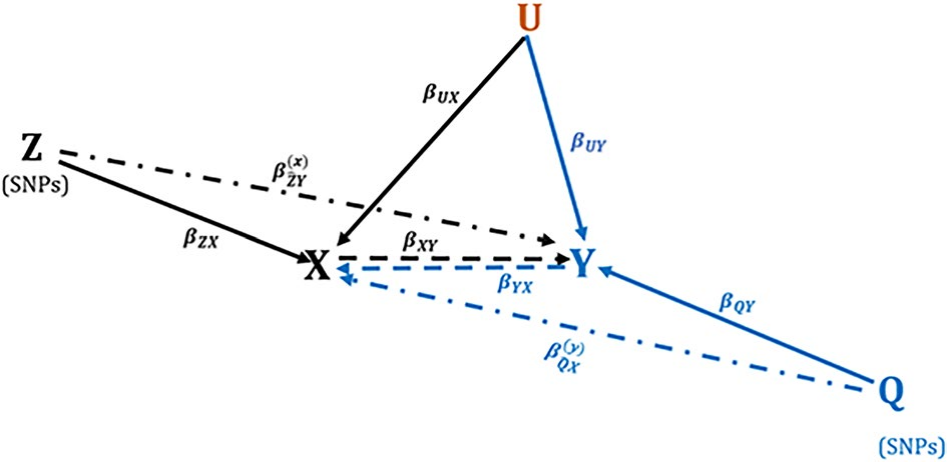
A graphical depiction of the complex relationship between relapses ($Y$) and worsening of disability ($X$). The parameters ${\beta }_{\widehat{Z}Y}^{(x)}$ and ${\beta }_{\widehat{Q}X}^{(y)}$ are the indirect contribution (residual effects) of the *WS-GPI* and *RS-GPI* on relapse risk and risk of worsening of disability. All other parameters represent direct associations.

The relationship between a relapse status ($$Y$$) and a disability worsening status ($$X$$) is graphically depicted on Fig. 2 (black arrows). In the first instance, let $$X$$ (a worsening status) be our exposure variable of interest, and let $$Y$$ (a relapse status) be the outcome of interest. We are interested in estimating the indirect contributions of a disability worsening status $$X$$ on a relapse status $$Y$$. The parameter representing this indirect association is given by $${\beta }_{\widehat{Z}Y}^{(x)}$$ (Fig. 2). The remaining parameters are defined as follows: $${\beta }_{ZX}$$ is the direct regression effect of genetic variants ($$Z)$$ on a worsening status $$X$$; $${\beta }_{XY}$$ is the direct regression effect of a worsening status $$X$$ on the relapse status $$Y$$, investigated before^20^; $${\beta }_{UY}$$ is the direct regression effects of potential clinical and environmental confounders ($$U)$$ known to predict relapse risk ($$Y$$), investigated previously^20^. To estimate indirect association parameter $${\beta }_{\widehat{Z}Y}^{(x)}$$, a three-stage statistical analysis procedure was employed as follows:

##### Stage 1: Constructing a time-dependent WS-GPI

As described in our previous analysis^20^, a Cox model was used to construct a time-dependent worsening-specific genetic prognostic index (*WS-GPI)* for the disability worsening outcomes status (our exposure variable $X$) by regressing $X$ on a set of MS related genetic variants $Z$. These genetic variants were selected using a penalised Cox model (described in Appendix 2)^59^. This model was fitted using the “*coxme*” R function^60^. Note that the combined effects of the genetic variants Z included in the WS-GPI is captured by the parameter ${\beta }_{ZX}$ defined above, and depicted on Fig. 2.

##### Stage 2: Predicting the longitudinal evolution of the WS-GPI

A Bayesian mixed-effect longitudinal sub-model (described in Appendix 3) was then used to estimate the non-linear subject-specific profiles (predicted values) of the *WS-GPI* over natural cubic splines of time with two internal knots placed at the 33rd and 66th percentile of the follow-up times. Boundary knots were set at 0.5 and 13 years.

##### Stage 3: Estimating the indirect association parameters

The final step was to estimate the association parameter ${\beta }_{\widehat{Z}Y}^{(x)}$ defining the indirect contributions of disability worsening outcomes to MS relapses. To achieve this, the predicted values of the *WS-GPI* obtained from stage 2 were then regressed against the relapse status ($Y$), using a univariate constant coefficient joint model (CCJM) (described in Appendix 3). In the CCJM, we adjusted for the effects of potential confounders shown to predict relapse risk in this cohort^20^. These included age at onset, sex, BMI, previous number of relapses (relapse counts), VitD status, and baseline T2L load. Smoking status was not included following its non-significant effects in predicting relapse risk. Because EDSS evolves continuously in time, we fitted flexible univariate varying coefficient joint models (VCJM)^61,62^, and compared their predictive accuracies with the univariable CCJM.

Because different features of the *WS-GPI* could indirectly influence the relapse-free survival process, we examined three hypotheses, each relating the indirect contributions (underlying residual effects) of the worsening status (captured in the *WS-GPI*) to the risk of relapses. Specifically, we hypothesised that relapse risk depends on the (1) *current value* (CV*)*, (2) *current value and slope* (CVS), and (3) *cumulative effects* (CE) of the predicted value of *WS-GPI*, respectively. To allow comparison between CCJM and VCJM, we approximated the baseline hazards of the CCJM with penalized P-splines^62^.

#### Analysis of time-to-worsening of disability

The analysis of the time-to-worsening of disability is the exact opposite and replica of the analysis of time-to-relapse described above. That is, we analysed the time-to-worsening of disability using the same methods but considering a reversed direction of the association between X and Y, represented by blue arrows on Fig. 2. In this analysis, the relapse status (*Y*) is now our exposure variable of interest, whereas the worsening status ($$X$$) is the outcome of interest. Similarly, we constructed a relapse-specific genetic prognostic index (*RS-GPI)* using a set of genetic variants ($$Q$$) that were predictive of the relapse status ($$Y$$), using the same Cox model (Appendix 2). The parameters of the blue arrows on Fig. 2 are defined as follows: $${\beta }_{QY}$$ is the direct regression effect of individual MS risk variants ($$Q)$$ on the relapse status $$(Y)$$; $${\beta }_{YX}$$ is the direct regression effect of a relapse status ($$Y)$$ on a worsening status ($$X$$); $${\beta }_{\widehat{Q}X}^{(y)}$$ is the indirect contributions of MS relapses on a disability worsening status $$X$$; $${\beta }_{UX}$$ is the direct regression effects of potential confounders (defined above) on the worsening status ($$X$$).

To estimate the indirect association parameters ${\beta }_{\widehat{Q}X}^{(y)}$, two statistical formulations in both the CCJM and VCJM were considered. In the first model, we posit that the risk of worsening depends on the CV, CVS, and the CE of the predicted values of the *RS-GPI* (Appendix 3). In the second model, we posit that the worsening risk depends on the 3- and 6-months’ time-lagged values of *RS-GPI*, achieved by lagging the actual survival times. Lagging the actual times 3- and 6-months to observing a worsening status reduces the direct influence of measured relapses on EDSS scores assessed during the relapse phase^5,18^. In this way, the analysis of the time-to-worsening of disability could be seen as the analysis of progression independent of relapse activity (PIRA)^15^.

#### Adjusting for (time-dynamic) treatment effects

To examine how the indirect associations between relapses and disability worsening outcomes could be modulated by DMT treatments, we included in the joint models interactions between the WS-GPI and RS-GPI with the duration of DMT use (see Appendix 3). That is, we estimated the association parameters for each DMT arms separately in the joint model. The DMT variables used were the duration of 1st-line therapy (interferons and glatiramer acetate), 2nd-line therapy (oral therapies teriflunomide, and dimethyl fumarate), and 3rd-line therapy (natalizumab, alemtuzumab, and fingolimod). As this cohort was recruited before routine use of 2nd and 3rd-line therapies, there were too few cases on 2nd line therapy to be included; and therefore, the time spent without medication (duration of disease without DMT) formed our reference category.

### Statistical inference and software

The Bayesian approach to estimation of joint models implemented in the “*JMBayes2*” Rpackage^63^ was used to analyse the data. Using Gibbs sampler, we set up three 3 independent Markov’s chains each of length 10,000, and sampled from a multivariate normal posterior. We used 5000 iterations as the burn-in part, and another 5000 for computing the posterior summaries and posterior densities. A normal independent prior distribution was assumed for fixed-effects regression parameters, while a gamma prior was used for the variance parameters. The Gelman-Robin’s diagnostics were used to assess model convergence. Briefly, we trained mixed-effects and survival models using observations at the current clinical visit (the current epoch) and used it to predict future outcomes. That is at each clinical visit, we fitted joint models (described above) and dynamically updated the predictions using future outcomes; and obtained individual survival probabilities in real-time. The posterior means and 95% credible intervals were used to ascertain the statistical significance. The time-dynamic area under the receiver operating characteristic curve (AUC(*t,* $\Delta t$)), deviance information criterion (DIC), and dynamic prediction errors ($\mathrm{PE}(t,\Delta t)$) were used to assess model performance. To compare models, we used follow-up times *t* = 2.5, 5, 7.5 years, and a prediction window of width $\Delta t$ =2.5 years.

## Results

### Cohort characteristics

Participants across the four centres had similar baseline characteristics. In the analysis cohort, the mean age at onset was 37 years (SD = 9 years). The cohort characteristics are given in Table 1. There were 471 validated worsening events and 856 relapses.

**Table 1:** Cohort characteristics, demographics, and follow-up times.

N = 279 subjects in the AUSLONG study, with 3065 repeated EDSS observations. N = 253 subjects included in the analysis, 2453 repeated EDSS observations. N_miss_ = 471 missing EDSS points that were omitted in the analysis. D_1_ = 481 valid worsening events. D_2_ = 856 relapsing events.		
		Overall data
Female/male	196/57	253
Mean age at onset female/male (yrs)	37.6/37.4	37.5
Median EDSS at onset female/male	2.5/2.0	2.5
Median EDSS at diagnosis female/male	3.5/2.5	3.0
Median EDSS at year 10 female/male	5.0/5.0	6.0
Average follow‐up from onset female/male (yrs)	8.8/8.2	8.5
Average follow‐up from diagnosis female/male (yrs)	7.1/7.9	7.5
Average time to first relapse female/male (mnths)	8.4/6.7	7.6
Mean T2L count at baseline female/male	8.5/8.0	8.3
Relapse rates per year female/male	0.2/0.3	0.3
Mean body mass index at baseline female/male	26.4/28.9	27.7
Mean body mass index at year 10 female/male	27.0/28.3	27.7
Average disease duration at year 10 female/male (yrs)	9.9/8.6	9.3

### The direct effects of genetic variants on relapses and disability worsening outcomes

The regression effects of the genetic variants $Z$ and $Q$ that were used to construct the *WS-GPI* and *RS-GPI* respectively, are given on Table 2. The final genetic models for each survival outcome included the effects of the primary signal (*chr6:32,413,545*) that maps to the *HLA-DRB1* gene (HLA-DRB1*15:01 allele; RefSNP: *rs3129889*) following its previously established primary role in MS susceptibility^64^. Additionally, 5 SNPs (chr5:55,444,683, chr10:94,479,107, chr4:109,058,718, chr20:44,734,310, chr6:33,047,466) were found to be predictive of relapse risk and risk of worsening of disability in varying magnitudes and direction. Because MS relapses not measured during EDSS visits were excluded, the number of genetic variants (Table 2) used to construct the RS-GPI and WS-GPI in this study ($n$=2453 visits) were slightly different from those presented in our previous analysis on the same cohort ($n$=2858 visits). However, the total number of participants ($n$=253) remained the same.

**Table 2:** Effects of MS related genetic markers on the risk of worsening of disability and relapses, respectively.

Analyses of time-to-worsening (N = 2453, D_1_ = 481)				Analyses of time-to-relapses (N = 2453, D_2_ = 856)			
Instruments:$Z$				Instruments:$Q$			
SNP (chr:pos)	A1/A2	HR (SE)	P-value	SNP (chr:pos)	A1/A2	HR (SE)	P-value
*2:61,242,410*	*A/G*	0.14 (0.35)	2.89e-08	*1:93,152,635*	*T/C*	0.74 (0.62)	2.17e-06
*12:111,884,608*	*T/C*	5.39 (0.34)	5.49e-07	*2:112,770,799*	*A/G*	1.33 (0.12)	1.44e-02
*6:31,322,522*	*A/C*	8.68 (0.40)	8.80e-08	*3:100,848,597*	*T/C*	2.03 (0.17)	1.64e-03
*18:56,348,044*	*G/A*	0.20 (0.37)	1.05e-05	*6:32,422,125*	*G/A*	36.97 (2.05)	3.59e-02
*8:144,986,793*	*T/C*	0.25(0.35)	9.17e-05	*9:100,868,189*	*C/T*	0.14 (0.69)	4.42e-03
*11:95,311,422*	*C/T*	0.13 (0.44)	2.17e-06	*10:31,395,761*	*A/G*	36.97 (0.58)	6.53e-10
***5:55,444,683***	***A/G***	**0.87 (0.06)**	**3.00e-02**	***5:55444683α***	***A/G***	**0.16 (0.09)**	**5.97e-02**
*3:71,535,338*	*G/T*	0.22 (0.33)	4.13e-06	*11:64,095,178*	*C/T*	1.22 (0.09)	2.86e-02
*7:27,135,314*	*C/T*	4.81 (0.43)	2.45e-04	*12:6,440,009*	*C/T*	0.75 (0.11)	6.59e-03
***10:94479107γ***	***G/A***	**0.96 (0.01)**	**3.45e-04**	***10:94479107γ***	***G/A***	**0.94 (0.02)**	**3.82e-04**
***10:94,479,107***	***G/A***	**3.31 (0.36)**	**9.77e-04**	***10:94,479,107***	***G/A***	**9.97 (0.57)**	**4.93e-05**
*6:31322522γ*	*A/C*	0.94 (0.01)	1.15e-07	*3:121542898α*	*T/C*	0.78 (0.08)	2.93e-03
*18:56348044γ*	*G/A*	1.04 (0.01)	1.31e-04	*16:30,103,160*	*A/C*	0.76 (0.09)	1.06e-03
***4:109,058,718***	***T/G***	**2.53 (0.34)**	**6.97e-03**	***4:109,058,718***	***T/G***	**0.64 (0.11)**	**6.02e-05**
***4:109058718γ***	***T/G***	**0.97 (0.01)**	**2.16e-03**	***4:109058718α***	***T/G***	**0.84 (0.09)**	**4.92e-02**
*7:128573967α*	*A/G*	0.77 (0.07)	3.47e-04	*6:31540757α*	*C/A*	0.76 (0.12)	2.09e-02
*1:85,729,820*	*G/A*	0.76 (0.09)	1.82e-03	*6:33,081,823*	*G/A*	1.25 (0.10)	2.53e-02
***6:32,413,545***	*G/A*	**0.96 (0.44)**	**9.35e-01**	***6:32,413,545***	***G/A***	**0.84 (2.00)**	**3.59e-01**
***6:32413545α***	*G/A*	**1.17 (0.13)**	**2.34e-01**	***6:32413545α***	***G/A***	**0.84 (0.27)**	**5.08e-01**
*4:48,127,262*	*G/A*	1.25 (0.06)	1.09e-04	***6:32413545γ***	***G/A***	**1.02 (0.05)**	**6.73e-01**
*2:61242410γ*	*A/G*	1.05 (0.01)	8.35e-08	*1:212877776α*	*A/G*	1.28 (0.08)	1.60e-03
*12:111884608γ*	*T/C*	0.96 (0.01)	2.28e-06	*2:12607893α*	*T/C*	0.77 (0.08)	5.53e-04
***20:44,734,310***	***C/T***	**0.72 (0.10)**	**7.68e-04**	***20:44,734,310***	***C/T***	**0.16 (0.60)**	**2.10e-03**
***20:44734310α***	***C/T***	**0.71 (0.13)**	**1.14e-02**	***20:44734310γ***	***C/T***	**1.05 (0.02)**	**3.01e-03**
*3:71535338γ*	*G/T*	1.05 (0.01)	5.25e-07	*5:133891282α*	*T/C*	0.80 (0.07)	3.15e-03
*8:144986793γ*	*T/C*	1.03 (0.01)	7.90e-04	*6:137438057α*	*A/G*	1.57 (0.10)	1.74e-05
*1:160,389,984*	*G/A*	0.43 (0.32)	8.78e-03	*14:103,265,844*	*A/G*	0.13 (0.57)	3.62e-04
*7:27135314γ*	*C/T*	0.95 (0.01)	4.47e-05	*19:10592144α*	*C/T*	1.70 (0.10)	3.78e-08
***6:33,047,466***	***C/T***	**1.15 (0.07)**	**4.62e-02**	***6:33047466α***	***C/T***	**0.79 (0.08)**	**4.83e-03**
*10:64449549α*	*T/G*	0.69 (0.13)	4.63e-03	*20:52789743α*	*G/C*	0.70 (0.06)	1.02e-08
*8:144986793α*	*T/C*	0.74 (0.11)	8.00e-03	*22:37258986α*	*T/C*	0.78 (0.07)	3.95e-04
*11:95311422γ*	*C/T*	1.05 (0.01)	1.45e-04	*6:31497244α*	*A/G*	0.73 (0.10)	1.64e-03
*5:158,759,900*	*A/G*	1.40 (0.09)	1.43e-04	*17:73,335,776*	*C/G*	0.78 (0.09)	6.64e-03
*6:32145399α*	*G/C*	1.79 (0.20)	3.68e-03	*10:81059335α*	*T/G*	0.77 (0.08)	7.50e-04
*11:95311422α*	*C/T*	0.73 (0.16)	4.52e-02	*10:31395761γ*	*A/G*	0.91 (0.02)	1.24e-08
*5:158759900α*	*A/G*	1.37 (0.11)	4.96e-03	*9:100868189γ*	*C/T*	1.06 (0.02)	2.70e-03
*6:32145399γ*	*G/C*	1.02 (0.01)	4.98e-03	*1:93152635γ*	*T/C*	1.06 (0.02)	2.67e-04
*16:11114512α*	*G/A*	1.22 (0.08)	9.79e-03	*14:103265844γ*	*A/G*	1.05 (0.02)	2.27e-03
				*11:128421175α*	*A/G*	1.22 (0.07)	7.57e-03
				*6:32422125γ*	*G/A*	0.93 (0.05)	1.44e-01
				*6:31539767γ*	*T/C*	0.93 (0.02)	1.20e-04
				*1:93152635α*	*T/C*	0.77 (0.10)	6.35e-03
				*3:100848597α*	*T/C*	2.23 (0.18)	5.67e-06
				*6:31,539,767*	*T/C*	9.87 (0.62)	2.30e-04
				*9:100868189α*	*C/T*	0.62 (0.12)	3.38e-05
				*12:6440009α*	*C/T*	0.71 (0.10)	4.82e-04
				*17:73335776α*	*C/G*	0.85 (0.08)	4.65e-02
				*6:32422125α*	*G/A*	1.75 (0.25)	2.31e-02

Estimates have been adjusted for logarithm of disease duration (log-time) and standardized latitudinal coordinates. These SNPs were then used to construct the **WS-GPI** and **RS-GPI,** respectively.

Overlapping SNPs are highlighted bold across the table.

*HR* hazard ratio, *N* sample size, *D* number of events.

γ: indicates interaction with standardised latitudinal coordinates (latitudinal effects).

α: indicates interaction with time (time-dynamic SNP effects).

### Model selection

We observed better discriminative capability and prediction accuracy for the VCJM compared to the CCJM (Table S1). Based on higher AUC and smaller PE values, the CVS models are preferred. Also, the VCJM is preferred over the CCJM in explaining the relapse dynamics. Figure S1 provides further evidence that favours the use of a VCJM over CCJM. In the CCJMs (CVS model), the model adjusting for the duration of DMT use is preferred (see DIC on Table S2). Table S3 gives estimates for the parameters describing the non-linear profile of the (*WS*)*RS-GPIs* over time, with good discriminative capabilities.

### The indirect contribution of disability worsening outcomes to relapse hazards

The association parameters relating the indirect effects of disability worsening outcomes (captured in WS-GPI) with the risk for relapse under the CCJM are shown in Table 3. We found positive associations between the current value and slope of WS-GPI and the risk for relapses. In order words, disability worsening outcomes significantly contributed to relapse risk each year (HR = 3.45, C.I 3.29–3.61), and persisted over time (*HR* = *3.34, *C.I* 2.90–3.86*), regardless of DMT treatments. Further, given the effects of the current value of WS-GPI, we observed a smaller effect of 1st-line DMT use (HR = 0.68, C.I 0.58–0.79) on reducing the relapse hazards compared to 3rd-line DMT use (HR = 0.37, C.I 0.32–0.44), respectively. However, given the rates of change of WS-GPI (the slopes), both treatments had equal magnitudes in reducing relapse hazards significantly (1st-line DMT: HR = 0.52, C.I 0.42–0.66; 3rd-line DMT: HR = 0.52, C.I 0.40–0.67).

**Table 3:** Results of the CCJM: Posterior means (Est) and 95% credible intervals (C.I.) for the parameters in the current value (CV) and current slope (CS) model.

Model components	Unadjusted model	DMT-adjusted model
Effects	Estimates (95% C.I)	Estimates (95% C.I.)
Analyses of time-to-relapses (N = 2453, D_2_ = 856 relapsing events)		
Clinical and environmental effects		
Sex [male]	− 0.471 (− 0.497; − 0.445)	− 0.186 (− 0.205; − 0.168)
Log (age) at onset	0.009 (0.008; 0.010)	− 0.015 (− 0.016; − 0.013)
Body mass index	− 0.114 (− 0.159; − 0.069)	0.374 (0.322; 0.426)
Relapse counts	0.312 (0.303; 0.320)	0.390 (0.379; 0.402)
T2 lesion load	0.182 (0.154; 0.210)	0.020 (0.009; 0.031)
Vitamin D supplementation [Yes]	− 0.180 (− 0.241, − 0.118)	− 0.170 (− 0.212; − 0.129)
Association parameters		
WS-GPI [value]	0.919 (0.892; 0.945)	1.086 (1.068; 1.104)
WS-GPI [slope]	0.895 (0.652; 1.138)	1.207 (1.063; 1.351)
WS-GPI [value] $\times$ DDMT (Cat. 1)		− 0.387 (− 0.542; − 0.233)
WS-GPI [slope] $\times$ DDMT (Cat. 1)		− 0.648 (− 0.879; − 0.417)
WS-GPI [value] $\times$ DDMT (Cat. 3)		− 0.984 (− 1.142; − 0.827)
WS-GPI [slope] $\times$ DDMT (Cat. 3)		1.207 (1.063; 1.351)
Analyses of time-to-worsening of disability (N = 2453, D_1_ = 481 worsening events)		
Clinical and environmental effects		
Sex [male]	− 1.908 (− 1.948; − 1.868)	− 1.431 (− 1.472; − 1.390)
Log (age) at onset	− 0.014 (− 0.015; − 0.013)	− 0.015 (− 0.015; − 0.014)
Body mass index	0.025 (0.022; 0.027)	0.018 (0.017; 0.020)
Relapse counts	0.080 (0.067; 0.094)	− 0.005 (− 0.014; 0.004)
T2 lesion load	0.949 (0.920; 0.977)	0.613 (0.587; 0.639)
Vitamin D supplementation [Yes]	− 0.051 (− 0.077; − 0.025)	− 0.081 (− 0.107; − 0.056)
Association parameters		
RS-GPI [value]	1.513 (1.436; 1.589)	1.238 (1.192; 1.284)
RS-GP [slope]	− 2.517 (− 3.259; − 1.774)	− 1.580 (− 1.880; − 1.280)
RS-GPI [value] $\times$ DDMT (Cat. 1)		− 0.302 (− 0.365; − 0.239)
RS-GP [slope] $\times$ DDMT (Cat. 1)		0.443 (− 0.151; 1.037)
RS-GPI [value] $\times$ DDMT (Cat. 3)		− 0.110 (− 0.165; − 0.055)
RS-GP [slope] $\times$ DDMT (Cat. 3)		0.365 (− 0.192; 0.922)

DDMT (Cat. 1): Duration of DMT category 1.

DDMTs (Cat. 3): Duration of DMT category 3.

WS-GPI [value]: Current value of the WS-GPI.

WS-GPI [slope]: Current slope of WS-GPI.

RS-GPI [value]: Current value of the RS-GPI.

RS-GPI [slope]: Current slope of RS-GPI.

**Figure 3:**
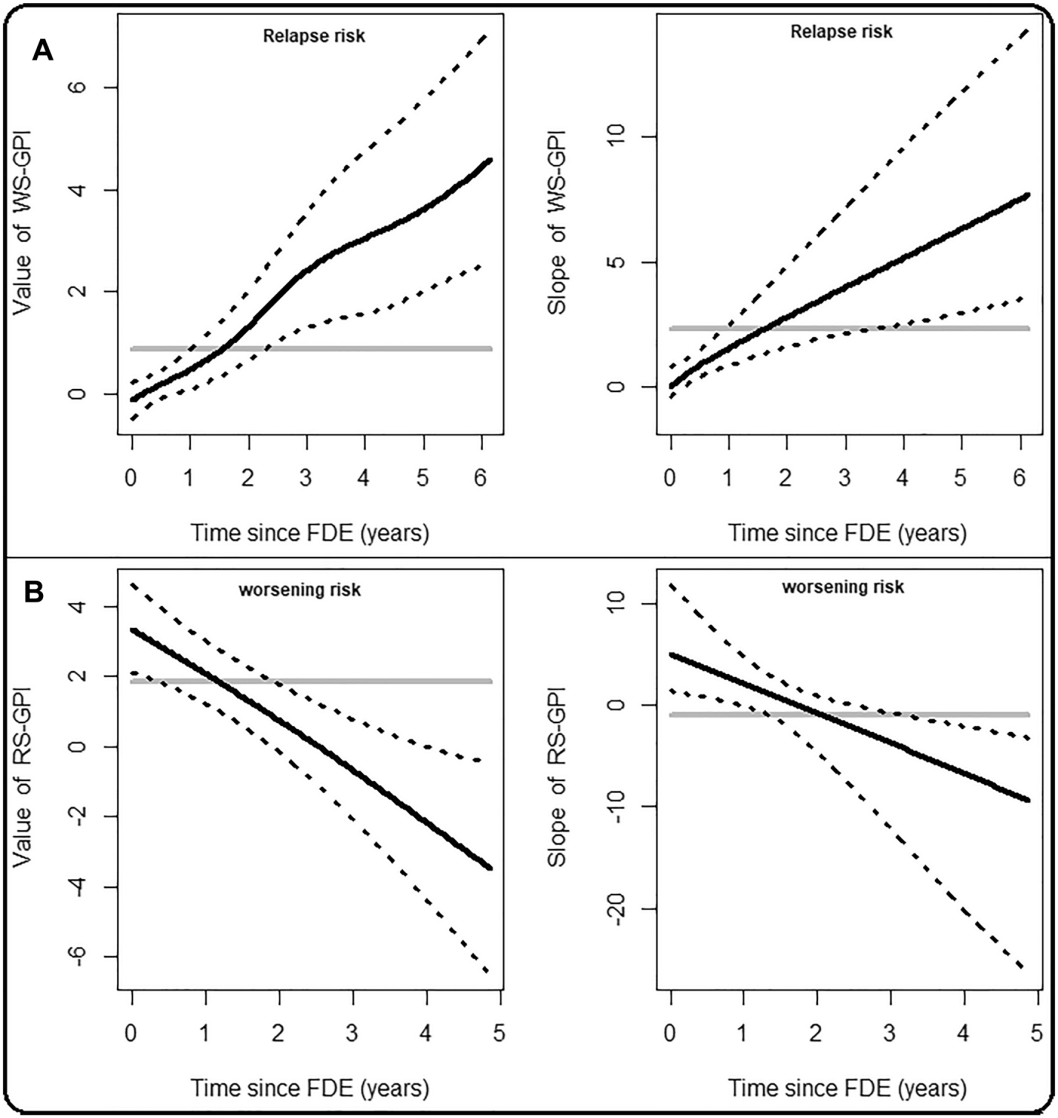
Time-dynamic associations between relapses and worsening outcomes in ROMS. The posterior means (black lines) and 95% credible intervals (dash lines) are time-varying estimates of association parameters obtained from the VCJM. The grey solid lines denote the time-fixed estimates of the association parameters obtained from CCJM.

Graphical estimates of the VCJM (Table S4) are shown on Fig. 3A. From onset (*t* = *0*), we observed non-linear effects for increasing values of the WS-GPI on the relapse-free survival, whereas the effect of the slope increased linearly with time. Specifically, 3 years post-onset, for individuals having the same sex, BMI score, T2L load, VitD status, relapse counts, and same value of WS-GPI at baseline, the log-hazard ratio for 1-unit increase in the slope of WS-GPI is 2.4. However, 6 years post-onset, this effect increases to 4.6. In sum, these results suggest that worsening outcomes are predictive of relapses (indirectly via the WS-GPI) and increases the risk of subsequent relapses with time. Furthermore, the longer the time spent on any DMT, the greater is the reduction in relapse risk (time-fixed effects in CCJM), and the risk of subsequent of relapses over time (time-varying effects in VCJM).

### The indirect contribution of relapses to disability worsening outcomes

There were no significant differences in the association parameters obtained before and after lagging the survival times suggesting no bias in the EDSS assessments that could be attributed to concurrent relapses (PIRA analysis). The results of the models without lagging the actual survival times are presented in Table 3. The current value and slope of the RS-GPI were both associated with the risk of worsening under the CCJM. Specifically, relapses accrued within 2.5 years of disease activity predicted short-term (< 2.5 years post-onset) disability worsening outcomes (HR = 3.45, C.I 2.29–3.61) per relapse, but did not contribute to long-term (> 2.5 years post-onset) disability worsening thereafter (HR = 0.21, C.I 0.15–0.28). Given the effects of the current value of RS-GPI, we observed a bigger effects of 1st-line DMT use (HR = 0.74, C.I 0.69–0.79) on reducing the worsening hazards compared to 3rd-line DMT use (HR = 0.90, C.I 0.85–0.95). However, given the rates of change of the RS-GPI (the slopes), the rates of disability accrual were not significantly modulated by DMT use (1st-line DMT: HR = 1.56, C.I 0.86–2.82; 3rd-line DMT use: HR = 1.44, C.I 0.83–2.51).

The time-dynamic effects of the current value and slope of the RS-GPI on the risk of worsening under the VCJM assumption are shown in Fig. 3B. Numerical estimates are shown in Table S5. Holding all other effects constant, the indirect contributions of relapses (captured in the current value of *RS-GPI*) have a decreasing impact on the worsening-free survival from onset (*t* = *0*) with an initial log-hazard of 3.4, trending towards the null (log-hazard≃0) at ≃2.5 years post-onset. Hereinafter, these effects diminished significantly with subsequent worsening outcomes over time. A similar trend is observed with the rates of change of the *RS-GPI* (slopes). The overall finding is that relapses contribute to short-term (< 2.5 years) worsening outcomes in ROMS indirectly through the *RS-GPI.* However, these effects diminished significantly with time, and became protective of worsening outcomes 2.5 years post-onset. In addition, DMTs use has a significant time-fixed (see *RS-GPI[Value]*$\mathrm{x}$* DDMT*; Table 3) effect on reducing the risk of disability worsening following the actions of relapses. However, the use of DMT on subsequent disability worsening due to relapses did not show any significant benefit (see *RS-GPI[Slope]*$\mathrm{x}$* DDMT*; Table 3).

### Person-specific dynamic time-course predictions

**Figure 4:**
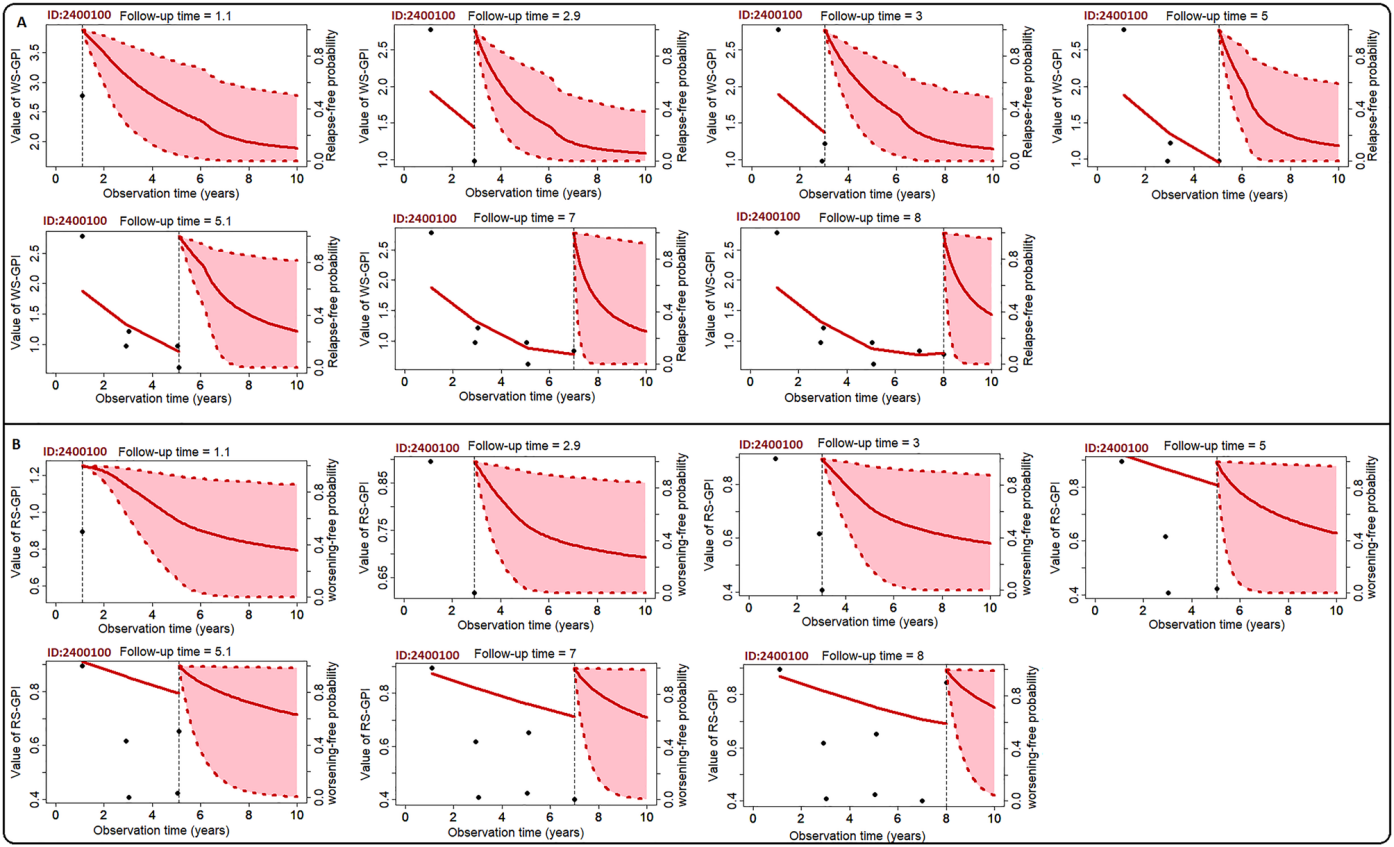
Real-time dynamic predictions for a 35-year-old male (AUSLONG ID = 2400100). (**A**). Time-dynamic relapse-free survival, (**B**). Time-dynamic worsening/disability-free survival. Vertical dotted lines denote the time point of the last EDSS measurement. On the left side, the fitted longitudinal trajectory of the WS-GPI (**A**) and RS-GPI (**B**) is presented (left of vertical line). On the right side, the solid lines represent the mean survival estimate, while dashed lines and shaded area are the corresponding 95% credible intervals.

In Fig. 4, we present real-time individualised predictions for a 35-years old male diagnosed with MS 7 years after his onset. Each time he was assessed, his relapse-free (Fig. 4A) and worsening-free (Fig. 4B) survival probabilities were updated simultaneously. Specifically, 2 years after observing his first value for *WS-GPI*, his relapse-free probability is ≃0.82, while 2 years after his last visit, this probability is ≃0.31. Conversely, his worsening-free probability (Fig. 4B) is ≃0.96 2 years after observing his first value for *RS-GPI*, and ≃0.71 2 years after observing the last value. Similar observations were made for the remaining participants in our study. These individualised predictions further confirm the observation that MS worsening outcomes contribute significantly to early (< 2.5 years post-onset) and future relapses, whereas relapses have little bearing on subsequent worsening outcomes. These results further suggest that subsequent worsening of disability in ROMS participants occurs in ways not clearly tied to relapses and depends on the previous worsening status. Overall, these findings translate to: *due to the effects of baseline MS risk variants, the underlying genetic burden of a worsening outcome (captured in the WS-GPI) significantly contributes to the subsequent recurrence of relapse activities in ROMS*.

## Discussion

We analysed a multicentre longitudinal prospective cohort of clinically isolated syndrome cases followed for more than 15 years with the majority converting to clinically definite MS in that time frame. We examined the complex time dynamic relationship between relapses and worsening of disability in MS using genetic prognostic indices predictive of these outcomes. Understanding the drivers of MS progression may provide potential insights to target MS treatments more accurately and effectively. We found that the effects of relapses on disability worsening outcomes, and vice-versa, were time dynamic. Relapses predicted worsening of disability in the early years of disease activity, but their longer-term impact on disability worsening outcomes diminished significantly with time. Conversely, worsening outcomes significantly increased relapse risk in the short (< 2.5 years post onset) and long-term (> 2.5 years post onset). These findings are in keeping with some but not all previous studies that have shown a non-sustained effect of relapses on disability worsening^12,14–20,22–24^.

Our study supports findings from recent works that use of DMTs delays disability accrual by years, with the potential to delay disability worsening being highest in the earliest stages of ROMS (< 2.5 years post-onset)^1,12,65^, and thus supports the commencement of DMTs during early years of disease activity. Further, jointly modeling the correlation between the longitudinal genetic prognostic indices with each survival outcome did enhanced the associations between these outcomes, compared to previous works^20^. Specifically, we observed enhancement in both magnitude and direction of the association in risk factors like age at onset, body mass index, relapse counts, and baseline T2 lesion load. Particularly, we found a significant positive effect of baseline T2 lesion load on relapse rates without adjusting for the duration of DMTs used. However, after adding interactions with the duration of DMTs used, the effects of baseline T2 lesion were borderline significant. Compared to previous findings^20^, we further observed a big positive effect of baseline T2 lesion load on worsening rates, before and after adjusting for the duration of DMTs use.

Following the results from our PIRA analysis (not shown), the data suggested that pre-existing disability and older age were principal risk factors for disability accumulation in the short-term (< 2.5 years post-onset), confirmed recently in Lublin et al.^1^ However, in terms of long-term worsening (> 2.5 years post-onset), the duration of DMTs use did not show any significant long-term benefits. Further, male sex, vitamin D3 supplementation status, and body mass index were significant predictors of relapses and worsening outcomes. Specifically, the relapse counts were significant predictors of relapse risk, but not risk of worsening. Compared to our previous analysis^20^, the latter results are novel and of interest. Nevertheless, these results do support previous findings from observational studies^66–69^ regarding the effects of DMTs in modulating relapse risk.

Relapses were significant predictors of short-term early disability worsening outcomes, but later relapses accrued 2.5 years post-onset did not contribute to further disability accumulation. In fact, their effects diminished significantly over time. Our results are not in line with early MS studies that showed that relapse frequency and incomplete recovery from relapses within the first few years of disease predicted short and long-term disability accrual^13,70–74^. However, our results suggests that this predictive effect is lost after 2.5 years of disease activity, and may interestingly reverse in direction thereafter. The current findings are further comparable to those from recent studies that reported relapses accrued early^1,11,12,14,15,22^, or within the first 2^1,12,22^ and 5 years^11,12^ of disease onset predicted short-term disability accumulation, and then lost their predictive value thereafter. This dissociating and negative impact of relapses on disability accumulation could be attributed to the increasing use of DMTs which directly suppresses relapses. Consequently, the natural decline in relapse rates over time may also mitigate any long-term relapse-associated worsening^12,27,75–77^. In other words, an increase in the use of DMT results in a decrease in relapse rates, which results in a decrease in the frequency of relapse-associated worsening outcomes. Therefore, the resulting negative association that we observed after 2.5 years of disease activity was due to direct effects of DMTs on relapse rates. This negative association of relapse activity on disability worsening outcomes could also be attributed to the fact that the relapse frequency in our data could have been underreported, as postulated Ahrweiller et al.^12^ We presented in Fig. 3B, graphical estimates of time-dynamic predictions that reveals the decreasing and dissociating impact of late relapses on disability accumulation. Based on these results, our finding that relapses accrued 2.5 years post-onset have no effect on long-term worsening outcomes, and may in fact mitigate against them, is novel and of interest.

Current disability worsening outcomes were associated with shorter time-to-relapse, and thus increased the risk associated with current and subsequent relapse activities, shown in Fig. 3A. However, these time-dynamic actions were not observed until, on average, 3 months after the first relapse phase has ended. Limited studies^1,12,15,18,21,78^ have investigated the *direct* impact of disability worsening outcomes on subsequent relapse status in ROMS. Our analysis is the first to examine the *indirect* effects of worsening outcomes on subsequent relapse risk. Our data suggested that future relapses occur as a results of the current disability worsening status, and that disability accumulation 2.5 years post-onset occurs in ways not tied to the current relapse status^18,21^.

### Clinical significance of the study

In the current study, DMTs were observed to be more effective in preventing short-term disability accumulation than they were at preventing long-term disability accumulation. This was due to their considerable influence on moderating the effects of both the current and subsequent relapse risks, the latter of which had a less marked effect on long-term disability accrual. Further, the occurrence of PIRA (despite effective DMTs) suggests that a gradual pathological process such as secondary degeneration (as a result of accumulating MRI brain lesions) play a key role in the accumulation of disability from disease onset. For instance, in the PARADIGMS study^79^, paediatric patients treated with interferon beta-1a or fingolimod experienced brain volume reduction rather than age-expected brain volume gain. Although fingolimod dramatically reduced the relapse frequency by 82% when compared to interferon beta-1a^80^, and significantly reduced the amount of brain volume loss, both active therapy groups showed a nett loss of brain volume. These findings, together with ours, imply that clinicians and clinical trials should aim at targeting both primary (e.g., glial and/or neuronal loss) and secondary (e.g. brain volume and/or new brain lesions) inflammations, as brain volume and neuronal loss appear to be common features in the early and latter stages of disease activity^81–83^.

Further, for real-time surveillance of a person’s disease progression status, we presented quantitative measures of both constant and time-dynamic associations in Fig. 3B, and individual time-dynamic survival probability estimates in Fig. 4A. These results, if externally validated, can be incorporated into software tools that provide vital information regarding a person’s future progression status. Further, clinicians could use these predictions, alongside recently validated genetic prediction rules^41^, and identify persons with ROMS at greater risk of disability accrual in the short and medium term, and initiate early treatment with DMTs, or institute more aggressive MS therapies where indicated.

### Strengths and limitations

Despite these interesting findings, the current study is not without limitations. Specifically, our study is limited by its observational non-randomised longitudinal cohort design. In this context, a longitudinal analysis using a 15-year cohort study does provide the best methodology to ascertain these associations as it would be unethical and infeasible to undertake a 15-year placebo-controlled intervention study. Additionally, there is also likely to be indication bias where higher efficacy DMTs are given to those with worse markers of disease activity. However, the availability of prospectively collected long-term data with repeated measures significantly enhances the power of this study. Other unmeasured biomarkers of disease activity and progression, particularly cerebrospinal fluid biomarkers of axonal damage, neuronal damage, glial dysfunction, demyelination, and inflammation^84,85^; and neurofilament light chains^84,86^ can be potentially explored to further enhance these associations.

Further, we did not adjust the association parameters for vitamin D3 supplementation status, as the effects of vitamin D3 supplementation in clinical trials has been underwhelming^29–31^. Specifically, the dosage of vitamin D3 supplements were not specified for most of our participants but largely consisted of vitamin D3 in multivitamin preparations and was in the range of 200-400 IU daily of vitamin D3. This dosage may not be sufficient to increase serum levels of vitamin D, and therefore its direct effect on relapse risk and risk of worsening should be interpreted with caution.

It is important to note that the models used to estimate the association parameters have no connection with mendelian randomisation (MR). As a result, the parameters describing the indirect associations are not causal effects estimates. In fact, a typical MR analysis could not be used in our study because it was difficult to determine a priori, the directions of the causal effects. MR analyses are mostly applied to baseline or cross-sectional slices of data and cannot be used to study the underlying longitudinal process governing the time-dynamic effects of RS-GPI and WS-GPI, respectively on relapse and worsening risk. Our study could be further enhanced by considering horizontal pleiotropy amongst the genetic variants being investigated.

The lack of an external validation cohort is another limitation of this study, and we are unaware of a similarly conducted prospective cohort with all investigated factors measured. However, similar findings from recent clinical studies^1,12,15,18,20,21^ with large sample sizes investigating the *direct* associations thus provides evidence that goes further to confirm the findings (*the indirect associations*) presented in this study. Additionally, our study further demonstrates real-world clinical application by providing unbiased estimates of individual real time survival probabilities for predicting future disability outcomes in people with ROMS.

## Conclusion

In summary, we examined the indirect contribution of relapses to disability worsening outcomes, and vice-versa, and provided robust measures of associations adjusted for the duration of treatment effects, and clinical and environmental predictors. In ROMS, relapses accrued within 2.5 years of MS onset are strong indicators of disability worsening, but late relapses accrued 2.5 years post onset are not overt risk factors for further disability worsening. In contrast, disability worsening outcomes are strong positive predictors of current and subsequent relapse risk. Long-term DMT use and older age strongly influence the individual outcomes and their associations.

## Supplementary Information

Supplementary Information 1.

Supplementary Information 2.

## Data Availability

For reproducibility, the R-codes and model output are given in the supplementary result file (Sect. “Introduction”). The AUSLONG data are not publicly available due to privacy and ethical restrictions but can be obtained from the AUSLONG Investigator group (https://www.msaustralia.org.au/ausimmune/) as part of bona fide research collaborations. The AUSLONG genotype data has been uploaded to dbGaP (https://www.ncbi.nlm.nih.gov/gap/) under accession: *phs000139.v1.p1*. Access to this data can be made via application to IMSGC^36^.
