# Supplementary material for: The association between disability progression, relapses, and treatment in early relapse onset MS: an observational, multi-centre, longitudinal cohort study
Source: Sci Rep. 2023 Jul 18;13:11584. doi: 10.1038/s41598-023-38415-z (PMC10354053; doi:10.1038/s41598-023-38415-z)
Supplement: Supplementary file 1 — Supplementary Information 1. [file 41598_2023_38415_MOESM1_ESM.pdf]

The association between disability progression, relapses, and treatment in early relapse onset MS: an observational, multi-centre, longitudinal cohort study.

Valery Fuh Ngwa
valeryfuh.ngwa@utas.edu.au

January 29, 2023

Appendix 1: Defining the Observation time for EDSS.

To acknowledge that disability accumulation on EDSS scale is a continuous-time process from onset of disease, we compute the observation time "Obstime" for the jth subject at the ith visit using the formular below.

$$\text{Obstime}_{\text{ij}} = \log(\sum_{i=1}^{m}[(t_i - DD_j) - (t_{i-1} - DD_j)])$$

$$\text{for } i = 1, \dots, n_i$$

$$\sum n_i = m, \qquad n = 1, \dots, 253, \qquad m = 2453$$

where t_i is the current EDSS visit date, t_{j-1} is the previous visit date, and DD_i is the date of diagnosis or date of first demyelinating events (FDEs), whichever comes first. That is for each subject, we took the cumulative sum of the difference in visit dates and adjusted for disease duration at the time of EDSS measurement.

Appendix 2: Constructing a time-dependent WS-GPI

To predict the risk of relapses, a Cox-Lasso model (1) was used to select the genetic variants (Z).

$$pl_{lasso}(\beta) = \sum_{j=1}^{n} \delta_j * \log\left(\frac{\exp(\beta Z_j^T)}{\sum_{i \in R(t_j)} \exp(\beta Z_j^T)}\right) - \frac{1}{\lambda}\sum_{j=1}^{p}|\beta_j|, \quad \lambda \geq 0 \qquad (1)$$

where Z_j is a fixed-effect genotype matrix (including interactions with time) representing the allele count for the jth individual, $pI_{lasso}(\beta)$ is the Cox-Lasso estimator for the partial log-likelihood of β, induced by the Breslow estimator log(.). Optimal values for penalty parameter λ were then chosen by leave-one-out cross-validation.

Using the selected variants (Z), the WS-GPI were constructed using model (2) as follows:

$$\mathrm{h_j}(\mathrm{t}|Z_j) = \mathrm{h_0}(\mathrm{t})\exp\left(\mathbf{Z}_\mathrm{j}^\mathrm{T}\boldsymbol{\beta}(\mathbf{t})\right) \quad (2)$$

$$\textit{WS-GPI}_j(t_w) = \log\left(-\log\left(\hat{\mathrm{S}}_{\mathbf{Z},\mathrm{CV,j}}(t_w)\right)\right)$$

where t_w is the EDSS observation time, and $\hat{S}_{\mathbf{Z},CV,\,j}(t_w)$ is the cross-validated predicted survival curve for jth individuals at time t_w.

Appendix 3: Predicting the longitudinal evolution of the WS-GPI.

A Bayesian mixed-effect longitudinal sub-model was then used to estimate the non-linear subject-specific profiles (predicted values) of the *WS-GPI* over natural cubic splines of the EDSS observation time t_w with two internal knots placed at the 33rd and 66th percentile of the follow-up times. Boundary knots were set at 0.5 and 13 years. In the model describing the longitudinal evolution of the *WS-GPI*, we posit that

$$\Omega_j(t) = \eta_j(t) + \varepsilon_j(t)$$
$$\eta_j(t) = \beta_0 + \textstyle\sum_{k=1}^{3}\beta_{1k}\{B_k(t_w,\lambda)\} + b_{j0} + \sum_{k=1}^{3} b_{jk}B_k(t_w,\lambda) \quad (3)$$
$$j = 1,\dots,n_{j.}$$

where $\boldsymbol{\Omega_i(t)}$ is the true value of the WS-GPI observed for the j-th individual at time t_w; $B_k(t_w,\lambda)$ is the natural cubic B-spline basis for time with two internal knots (λ) at 0.5 and 13 years (corresponding to 33rd and 66th percentile of the observed follow-up times); $\boldsymbol{\eta_i(t)}$ is the mean evolution of the *WS-GPI*, $b_{j0} \sim N(0,\sigma_{b0}^2)$ are normal random deviations from the overall mean β_0; $b_{jk} \sim N(0,\sigma_b^2)$ are normal random deviations from the overall slope β_{1k}; and $\varepsilon_i(t) \sim N(0,\sigma_e^2)$ are random errors assumed to be independent and normally distributed.

Appendix 4: Estimating the association parameters relating the effects of the *WS-GPI* to risk for relapses.

Joint Model Formulation

To compute the association estimates $\beta_{\hat{Z}Y}^{(x)}$ in the third stage, we postulate constant-coefficient joint models (CCJM) [32], and compared their predictive performance with varying-coefficient joint models (VCJM) [27]. Inspired by previous works (not related to MS) [26, 27, 30, 33], where different features of WS-GPI may influence the relapse-free survival process, we hypothesised that the underlying history of the WS-GPI namely: current value, current slope, and cumulative effects (area under profile), were each associated with the risk of relapses. In the CCJM, the survival submodels took the forms:

1. Current value of WS-GPI with no DMT adjustment

$$h_j(t_{r,}\boldsymbol{\theta}_s) = h_0(t_r)\exp\{\boldsymbol{\gamma U_j} + \alpha_1\boldsymbol{\eta_j}(t) + b_j\Gamma_j^T\} \tag{4}$$

2. Current value of WS-GPI with DMT interaction

$$h_j(t_{r,}\boldsymbol{\theta}_s) = h_0(t_r)\exp\{\boldsymbol{\gamma U_j} + \alpha_1\boldsymbol{\eta_j}(t) * DDMT_j + b_j\Gamma_j^T\} \tag{5}$$

3. Current value and slope of WS-GPI with no DMT adjustment

$$h_j(t_{r,}\boldsymbol{\theta}_s) = h_0(t_r)\exp\left\{\boldsymbol{\gamma U_j} + \alpha_1\boldsymbol{\eta_j}(t) + \alpha_2\frac{d\boldsymbol{\eta_j}(t)}{dt} + b_j\Gamma_j^T\right\} \tag{6}$$

4. Current value and slope of WS-GPI with DMT interaction

$$h_j(t_{r,}\boldsymbol{\theta}_s) = h_0(t_r)\exp\left\{\boldsymbol{\gamma U_j} + \alpha_1\boldsymbol{\eta_j}(t) * DDMT_j + \alpha_2\frac{d\boldsymbol{\eta_j}(t)}{dt} * DDMT_j + b_j\Gamma_j^T\right\} \tag{7}$$

5. Cumulative effects of the WS-GPI with no DMT adjustment

$$\mathrm{M}_{30}: h_j\left(t_{r,}\boldsymbol{\theta}_s\right) = h_0(t_r)\exp\left\{\boldsymbol{\gamma}\boldsymbol{U_j} + \alpha_3 \int_0^t \boldsymbol{\eta_j}(s)ds \ + b_j\Gamma_j^T\right\} \quad (8)$$

6. Cumulative effects of the WS-GPI with DDMT interaction

$$h_j\left(t_{r,}\boldsymbol{\theta}_s\right) = h_0(t_r)\exp\left\{\boldsymbol{\gamma}\boldsymbol{U_j} + \alpha_3 \int_0^t \boldsymbol{\eta_j}(s)ds * DDMT_j + b_j\Gamma_j^T\right\} \quad (9)$$

where $\boldsymbol{\gamma}$ is a vectors of fixed-effects regression coefficients with a corresponding vector of clinical and environmental covariates U_j; $\boldsymbol{t_r}$ is the time of relapse, $\boldsymbol{t}$ is the median of the relapse and worsening time (i.e. median of $\boldsymbol{t_w}$, and $\boldsymbol{t_r}$), $\boldsymbol{\theta}_s^T$ is a parameter vector of the relapse survival process; and $\boldsymbol{\alpha}_{(.)}$ are association parameters for the indirect contributions of worsening of disability (via the WS-GPI) to the relapse-free survival process ($\beta_{\hat{Z}Y}^{(x)}$), Γ_j^T is a vector of random effects with corresponding vector of random effects coefficients b_j. The random effects components included subject identifiers (IDs) nested within study site. Because the relapse times may differ from worsening times (Obstime), we opted to use the median time $\boldsymbol{t}$ between these endpoints to compute the association parameters.

Please note that the processes describing the current value, slope and cumulative effects of the WS-GPI are represented by the superscript "x" in the association parameter $\beta_{\hat{Z}Y}^{(x)}$ in Figure 2. Further, the parameter $\hat{z}$ in $\beta_{\hat{Z}Y}^{(x)}$ denotes the fitted profiles (predicted values) of the *WS-GPI* obtained from stage 2.
