# Supplementary material for: The association between disability progression, relapses, and treatment in early relapse onset MS: an observational, multi-centre, longitudinal cohort study
Source: Sci Rep. 2023 Jul 18;13:11584. doi: 10.1038/s41598-023-38415-z (PMC10354053; doi:10.1038/s41598-023-38415-z)
Supplement: Supplementary file 2 — Supplementary Information 2. [file 41598_2023_38415_MOESM2_ESM.pdf]

The association between disability progression, relapses, and treatment in early relapse onset MS: an observational, multi-centre, longitudinal cohort study.

Valery Fuh Ngwa
valeryfuh.ngwa@utas.edu.au

January 29, 2023

1 R-Codes for fitting a current value and slope CCJM adjusted for VitD and DDMT

This gives the estimates presented on Table 1 (see ”VitDxDDMT-adjusted” column)

```
#####################################################
##Step 1: Fit a Bayesian Linear Mixed model to
##       predict the evolution of WS-GPI and RS-GPI
####################################################

###Specify Cubic Spline parameters
(qt1=quantile(xLong$Obstime, c(0.33, 0.66)));range(xLong$Obstime)
w0<-c("ns(Obstime, 3)", "(ns(Obstime,3)|auslongid)" )
r0<-c("ns(Obstime, 3)", "(ns(Obstime,3)|auslongid)" )

##Specify response variables
####################
resvar1<-"WS-GPI"
resvar2<-"RS-GPI"

##Model formulas
formW<-as.formula(paste(resvar1, paste(w0, collapse=" + "), sep=" ~"))
formR<-as.formula(paste(resvar2, paste(r0, collapse=" + "), sep=" ~"))

###Set option for parallel processing
options(mc.cores = parallel::detectCores()-2)
```

```
### Fit models
lmeFitW0<- mvglmer(list(formW),
                   data = xLong,
                   families = list(gaussian),engine = "JAGS")
lmeFitR0<- mvglmer(list(formR),
                   data = xLong,
                   families = list(gaussian),engine = "JAGS")

#######################################################
### Step 2: Fit Robust Anderson-Gill Survival submodels
##          RRE = relapse status
##          WoD = worsening status
#######################################################

CoxREL <- coxph(Surv(timeR, RRE)~ Sex+I(Age)+BMI+Relcount+T2L+VitD+cluster(site),
                                   robust = TRUE, data = xSurv,
                                   model = TRUE, x=TRUE)

CoxWDS <- coxph(Surv(timeW, WoD)~ Sex+I(Age)+BMI+Relcount+T2L+VitD+cluster(site),
                                  robust = TRUE, data = xSurv,
                                  model = TRUE, x=TRUE)

###############################################################
### Step 3: Fit CCJM models to estimate association parameters
###############################################################

##Define derivative forms
dForm1 <- list("WS-GPI" = list(name="value"),
               "WS-GPI" = list(name="slope",
                          fixed  = ~ -1 + dns(Obstime, 3),
                          indFixed=2:4,
                          random = ~ -1 + dns(Obstime, 3),
                          indRandom=1:3))

dForm2 <- list("RS-GPI" = list(name="value"),
               "RS-GPI" = list(name="slope",
                          fixed  = ~ -1 + dns(Obstime, 3),
                          indFixed=2:4,
                          random = ~-1 + dns(Obstime, 3),
                          indRandom=1:3))
```

```
### First fit Current value model only

JMFitR.cv<-mvJointModelBayes(lmeFitW0, CoxREL, timeVar="Obstime") ##relapse risk
JMFitW.cv<-mvJointModelBayes(lmeFitR0, CoxWDS, timeVar="Obstime") ##worsening risk

### Now fit current value and slope model by updating the current value models

JMFitR.cvs <- update(JMFitR.cv, Formulas = dForm1) ##relapse risk
JMFitW.cvs <- update(JMFitW.cv, Formulas = dForm2) ##worsening risk

### Lastly, add interaction between the (RS)WS-GPI and DDMT
############################################################
### DDMTC1: 1 = Yes, 0 = No
### DDMTC3: 1 = yes, 0 = No
#############################################

IntW<-list("WS-GPI_value" = ~DDMTC1+DDMTC3, "WS-GPI_slope" =~DDMTC1+DDMTC3)
IntR<-list("RS-GPI_value" = ~DDMTC1+DDMTC3, "RS-GPI_slope" =~DDMTC1+DDMTC3)

##set priors for shrinking

priors = list(shrink_alphas = TRUE, shrink_gammas = TRUE)

##Now update the Current value and slope models

JMFitR.cvs.dmt<-update(JMFitR.cvs, Interactions=IntW, priors = priors) ##relapse risk
JMFitW.cvs.dmt<-update(JMFitW.cvs, Interactions=IntR, priors = priors) ##worsening risk

##########################################################
## Posterior summary measures shown on the Table 3
## Current Value + Slope with no DMT adjustment
## Survival process: Using WS-GPI to predict Relapse risk
##########################################################
>summary(JMFitW.cvs)

Call:
mvJointModelBayes(mvglmerObject = lmeFitW0, survObject = CoxREL,
    timeVar = "Obstime", Formulas = dForm1)

Data Descriptives:
Number of Groups: 253 Number of events: 43 (17%)
Number of Observations:
```

```
  biomrkW: 2453

Random-effects covariance matrix:
         StdDev    Corr
(I)1     0.6307  (Int)1 n(O,3)1 n(O,3)2
n(O,3)1  0.2146 -0.8093
n(O,3)2  1.2416 -0.9236  0.7734
n(O,3)3  0.2570 -0.8214  0.7509  0.8330

Survival Outcome: RRE
              PostMean  StDev  StErr    2.5%   97.5% P
SexMale        -0.4707 0.4185 0.0132 -0.4967 -0.4446 0
I(Age)          0.0091 0.0171 0.0005  0.0082  0.0101 0
BMI            -0.1136 0.9517 0.0229 -0.1586 -0.0686 0
RelCont         0.3117 0.1879 0.0044  0.3030  0.3203 0
T2L             0.1819 0.3363 0.0142  0.1539  0.2099 0
VitD[Yes]      -0.1800 0.2311 0.0310 -0.2410 -0.1184 0
biomrkW_value   0.9187 0.4835 0.0135  0.8921  0.9452 0
biomrkW_slope   0.8952 4.5345 0.1234  0.6522  1.1383 0

Longitudinal Outcome: biomrkW (family = gaussian, link = identity)
                PostMean  StDev  StErr    2.5%   97.5% P
(Intercept)       1.6830 0.0533 0.0017  1.5798  1.7888 0
ns(Obstime, 3)1  -0.5801 0.0315 0.0017 -0.6403 -0.5176 0
ns(Obstime, 3)2  -1.6312 0.1217 0.0041 -1.8710 -1.3958 0
ns(Obstime, 3)3  -0.5588 0.0535 0.0032 -0.6655 -0.4559 0
sigma             0.3019 0.0050 0.0002  0.2919  0.3115 0

MCMC summary:
iterations: 5000
burn-in: 5000
thinning: 1
time: 3.5 min

##########################################################
## Posterior summary measures shown on the Table 3
## Current Value + Slope with DMT adjustment
## Survival process: Using WS-GPI to predict Relapse risk
##########################################################
>summary(JMFitW.cvs.dmt)

Call:
mvJointModelBayes(mvglmerObject = lmeFitW0, survObject = CoxREL,
    timeVar = "Obstime", Formulas = dForm1, Interactions = Int3,
    priors = priors)
```

```
Data Descriptives:
Number of Groups: 253 Number of events: 43 (17%)
Number of Observations:
  biomrkW: 2453

Random-effects covariance matrix:
         StdDev    Corr
(I)1     0.6307  (Int)1 n(O,3)1 n(O,3)2
n(O,3)1  0.2146 -0.8093
n(O,3)2  1.2416 -0.9236  0.7734
n(O,3)3  0.2570 -0.8214  0.7509  0.8330

Survival Outcome: RRE
                       PostMean  StDev  StErr    2.5%   97.5%     P
SexMale                 -0.1864 0.2797 0.0094 -0.2049 -0.1678 0.000
I(Age)                  -0.0145 0.0155 0.0005 -0.0156 -0.0134 0.000
BMI                      0.3738 0.5049 0.0263  0.3220  0.4255 0.000
RelCont                  0.3902 0.1716 0.0059  0.3786  0.4018 0.000
T2L                      0.0199 0.1823 0.0058  0.0085  0.0313 0.001
VitD[Yes]               -0.1700 0.2310 0.0211 -0.2117 -0.1286 0.000
biomrkW_value            1.0864 0.3271 0.0091  1.0684  1.1045 0.000
biomrkW_value:DMTDTxC1  -0.3872 2.4801 0.0784 -0.5417 -0.2327 0.000
biomrkW_value:DMTDTxC3  -0.9844 2.5295 0.0800 -1.1420 -0.8268 0.000
biomrkW_slope            1.2070 2.3183 0.0733  1.0626  1.3514 0.000
biomrkW_slope:DMTDTxC1  -0.6478 3.9787 0.1172 -0.8787 -0.4168 0.000
biomrkW_slope:DMTDTxC3  -0.6587 4.0976 0.1296 -0.9140 -0.4034 0.000

Longitudinal Outcome: biomrkW (family = gaussian, link = identity)
                PostMean  StDev  StErr    2.5%   97.5% P
(Intercept)       1.6830 0.0533 0.0017  1.5798  1.7888 0
ns(Obstime, 3)1  -0.5801 0.0315 0.0017 -0.6403 -0.5176 0
ns(Obstime, 3)2  -1.6312 0.1217 0.0041 -1.8710 -1.3958 0
ns(Obstime, 3)3  -0.5588 0.0535 0.0032 -0.6655 -0.4559 0
sigma             0.3019 0.0050 0.0002  0.2919  0.3115 0

MCMC summary:
iterations: 5000
burn-in: 5000
thinning: 1
time: 4.6 min

#############################################################
## Posterior summary measures shown on the Table 3
## Current Value + Slope with no DMT adjustment
## Survival process: Using RS-GPI to predict worsening risk
#############################################################
```

```
> summary(JMFitR.cvs)

Call:
mvJointModelBayes(mvglmerObject = lmeFitR0, survObject = CoxWDS,
    timeVar = "Obstime", Formulas = dForm2)

Data Descriptives:
Number of Groups: 253 Number of events: 44 (17.4%)
Number of Observations:
  biomrkR: 2453

Random-effects covariance matrix:
         StdDev    Corr
(I)1     0.3838  (Int)1 n(O,3)1 n(O,3)2
n(O,3)1  0.1640 -0.1778
n(O,3)2  0.6543 -0.3829  0.0814
n(O,3)3  0.3282 -0.0674 -0.1567 -0.0186

Survival Outcome: WoD
              PostMean  StDev  StErr    2.5%   97.5% P
SexMale        -1.9082 0.6361 0.0202 -1.9480 -1.8684 0
I(Age)         -0.0140 0.0152 0.0005 -0.0150 -0.0130 0
BMI             0.0246 0.0248 0.0012  0.0223  0.0270 0
RelCont         0.0802 0.2667 0.0069  0.0665  0.0939 0
T2L             0.9487 0.4123 0.0145  0.9201  0.9773 0
VitD[Yes]      -0.0510 0.1310 0.0132 -0.0773 -0.0254 0
biomrkR_value   1.5126 0.9396 0.0388  1.4360  1.5891 0
biomrkR_slope  -2.5165 7.9384 0.3768 -3.2588 -1.7743 0


Longitudinal Outcome: biomrkR (family = gaussian, link = identity)
                PostMean  StDev  StErr    2.5%   97.5%     P
(Intercept)       1.1834 0.0736 0.0043  1.0444  1.3305 0.000
ns(Obstime, 3)1  -0.1998 0.0647 0.0062 -0.3245 -0.0708 0.004
ns(Obstime, 3)2  -0.2479 0.1875 0.0129 -0.6253  0.1108 0.186
ns(Obstime, 3)3   0.0028 0.1088 0.0084 -0.2210  0.2050 0.938
sigma             0.7249 0.0114 0.0004  0.7026  0.7464 0.000

MCMC summary:
iterations: 5000
burn-in: 5000
thinning: 1
time: 3.5 min

#############################################################
## Posterior summary measures shown on the Table 3
```

```
## Current Value + Slope with DMT adjustment
## Survival process: Using RS-GPI to predict worsening risk
#############################################################
> summary(JMFit2.cvs.dmt)

Call:
mvJointModelBayes(mvglmerObject = lmeFitR0, survObject = CoxWDS,
    timeVar = "Obstime", Formulas = dForm2, Interactions = Int4,
    priors = priors)

Data Descriptives:
Number of Groups: 253 Number of events: 44 (17.4%)
Number of Observations:
  biomrkR: 2453

Random-effects covariance matrix:
         StdDev    Corr
(I)1     0.3838  (Int)1 n(O,3)1 n(O,3)2
n(O,3)1  0.1640 -0.1778
n(O,3)2  0.6543 -0.3829  0.0814
n(O,3)3  0.3282 -0.0674 -0.1567 -0.0186

Survival Outcome: WoD
                       PostMean  StdDev  StErr    2.5%   97.5%     P
SexMale                 -1.4307 0.6606 0.0209 -1.4719 -1.3896 0.000
I(Age)                  -0.0148 0.0135 0.0004 -0.0155 -0.0140 0.000
BMI                      0.0183 0.0234 0.0007  0.0169  0.0198 0.000
RelCont                 -0.0048 0.1492 0.0047 -0.0141  0.0045 0.310
T2L                      0.6134 0.3906 0.0132  0.5874  0.6394 0.000
VitD[Yes]               -0.0813 0.1411 0.0131 -0.1070 -0.0556 0.000
biomrkR_value            1.2382 0.7051 0.0235  1.1920  1.2844 0.000
biomrkR_value:DMTDxC1   -0.3022 1.0079 0.0319 -0.3649 -0.2394 0.000
biomrkR_value:DMTDxC3   -0.1104 0.9246 0.0279 -0.1654 -0.0554 0.000
biomrkR_slope           -1.5799 4.8169 0.1523 -1.8799 -1.2798 0.000
biomrkR_slope:DMTDTxC1   0.4427 9.5341 0.3015 -0.1513  1.0366 0.142
biomrkR_slope:DMTDTxC3   0.3653 8.9423 0.2828 -0.1918  0.9223 0.196

Longitudinal Outcome: biomrkR (family = gaussian, link = identity)
                PostMean  StdDev  StErr    2.5%   97.5%     P
(Intercept)       1.1834 0.0736 0.0043  1.0444  1.3305 0.000
ns(Obstime, 3)1  -0.1998 0.0647 0.0062 -0.3245 -0.0708 0.004
ns(Obstime, 3)2  -0.2479 0.1875 0.0129 -0.6253  0.1108 0.186
ns(Obstime, 3)3   0.0028 0.1088 0.0084 -0.2210  0.2050 0.938
sigma             0.7249 0.0114 0.0004  0.7026  0.7464 0.000

MCMC summary:
```

```
iterations: 5000
burn-in: 5000
thinning: 1
time: 4.5 min
```

2 Additional Tables and Figures

Table S1. Cross-validated dynamic AUC and prediction errors (PE) for the CCJM and VCJM for different functional forms. Performance was assessed at median follow-up times t = 2.5, 5, and 7.5years, and a medically-relevant window of 2.5years. The results are based on a 5 fold Monte-Carlo cross-validation repeated 20 times.

		**CCJM**		**VCJM**	
t (yrs)	**Functional Forms**	**AUC(t+2.5)**	**PE(t+2.5)**	**AUC(t+2.5)**	**PE(t+2.5)**
**Prediction of relapse risk**					
**2.5**	Value	0.6523	0.2213	0.8352	0.2066
	Value + Slope	0.6685	0.1183	0.9291	0.1086
	Cumulative effects	0.6313	0.3235	-	-
**5**	Value	0.7024	0.2139	0.8853	0.1178
	Value + Slope	0.7245	0.1090	0.9851	0.0534
	Cumulative effects	0.6697	0.4167	-	-
**7.5**	Value	0.7335	0.1598	0.9164	0.0913
	Value + Slope	0.6954	0.1019	0.9560	0.0659
	Cumulative effects	0.6274	0.3410	-	-
**Prediction of Worsening of disability.**					
**2.5**	Value	0.7570	0.2278	0.7899	0.1792
	Value + Slope	0.7656	0.2277	0.8073	0.1798
	Cumulative effects	0.7289	0.2747	-	-
**5**	Value	0.7932	0.2254	0.8493	0.1406
	Value + Slope	0.8020	0.2001	0.8587	0.1267
	Cumulative effects	0.7376	0.2426	-	-
**7.5**	Value	0.8078	0.1505	0.9080	0.1078
	Value + Slope	0.8129	0.1152	0.9152	0.0987
	Cumulative effects	0.7252	0.1513	-	-

NB: Since the cumulative effects functional form is not available under the VCJM, we considered only the value and value + slope functional forms.

Table S2. Selection of the mean component of the CCJM based on marginal and conditional DIC. The model with best fit has the smallest DIC value

Associations:	*WS-GPI↔Relapse risk*		*RS-GPI↔Worsening risk*	
**Model Info**	**Marginal DIC**	**Conditional DIC**	**Marginal DIC**	**Conditional DIC**
Unadjusted	2148.016	951.915	5896.963	4912.357
VitD-adjusted	2147.591	934.046	5865.479	4797.130
DDMT-adjusted	2150.751	961.902	5890.886	4843.224
VitD×DDMT-adjusted	2145.693	946.708	5832.531	4734.383

Table S3. Posterior means and 95% credible intervals (C.I) for the parameters describing the evolution of WS-GPI and RS-GPI non-linear cubic splines function of the follow-up time [ns(time, 3)] in the Bayesian mixed effects model.

**Longitudinal process**				
	**WS-GPI ~ ns(time, 3)**		**RS-GPI ~ ns(time, 3)**	
**Effects**	**Estimate**	**95% C.I**	**Estimate**	**95% C.I**
Intercept	1.683	(1.580; 1.789)	1.183	(1.044; 1.331)
ns(time, 3)1	-0.580	(-0.640; -0.518)	-0.200	(-0.325; -0.071)
ns(time, 3)2	-1.631	(-1.871; -1.396)	-0.248	(-0.625; 0.111)
ns(time, 3)3	-0.559	(-0.665; -0.456)	0.003	(-0.221; 0.205)
Error (σ)	0.302	(0.292; 0.312)	0.725	(0.703; 0.746)
σ_{b0}	0.631		0.384	
σ_{b11}	0.215		0.164	
σ_{b21}	1.215		0.654	
σ_{b31}	0.257		0.328	
ICC	0.886		0.678	
pD	951.356		548.398	
DIC	2034.825		5928.552	
$\overline{D(\theta)}$	1083.469		5380.154	
$D(\widehat{\theta})$	132.113		4831.756	
$l(\theta)$	7875.074		14050.700	

WS-GPI: Observed value of the worsening-specific genetic prognostic index
RS-GPI: Observed values of relapse-specific genetic prognostic index.
ns(time,3): Non-linear spline function on the follow-up time.
ICC=Intra-class correlation; ***pD*** =effective degrees of freedom.
DIC= Deviance information criterion; $\sigma_{(.)}$= Variance of the random effects.
$\overline{D(\theta)}$ and $D(\widehat{\theta})$ are deviance parameters; and $l(\theta)$ is the log-likelihood.

Table S4. VCJM results: Using the current value and current slope of the WS-GPI to predict the risk for relapse. Posterior means, 95% credible intervals and Gelman-Rubin's diagnostic (Rhat = potential scale reduction factor), are shown.

	**Est**	**SE**	**2.5%**	**97.5%**	**Rhat**
Longitudinal process: WS-GPI ~ns(time, 3)					
Intercept	1.683	0.053	1.580	1.789	1.00
ns(time, 3)1	-0.580	0.032	-0.640	-0.518	1.00
ns(time, 3)2	-1.631	0.122	-1.871	-1.396	1.00
ns(time, 3)3	-0.559	0.054	-0.665	-0.456	1.00
Error (σ)	0.302	0.005	0.292	0.312	1.00
Survival process: WS-GPI ↔Relapse risk					
Sex(Male)	-0.319	0.012	-0.342	-0.296	1.00
Age at FDE	0.002	0.001	0.000	0.004	1.00
Body mass index	0.627	0.014	0.600	0.654	1.00
Relapse counts	0.252	0.005	0.242	0.262	1.00
T2 lesion counts	0.167	0.009	0.149	0.185	1.00
WS-GPI_Value[1]	-0.450	0.297	-1.029	0.129	1.00
WS-GPI_Value[2]	0.111	0.119	-0.121	0.343	1.00
WS-GPI_Value[3]	0.514	0.203	0.118	0.910	1.00
WS-GPI_Value[4]	1.161	0.306	0.565	1.761	1.00
WS-GPI_Value[5]	2.157	0.497	1.188	3.126	1.00
WS-GPI_Value[6]	2.796	0.676	1.478	4.114	1.00
WS-GPI_Value[7]	3.179	0.800	1.619	4.739	1.00
WS-GPI_Value[8]	3.621	0.818	2.025	5.216	1.00
WS-GPI_Value[9]	4.268	0.928	2.458	6.078	1.00
WS-GPI_Value[10]	5.064	1.192	2.740	7.388	1.00
WS-GPI_Slope[1]	-0.910	0.189	-1.279	-0.541	1.00
WS-GPI_Slope[2]	0.684	0.213	0.269	1.099	1.00
WS-GPI_Slope[3]	1.702	0.364	0.992	2.412	1.00
WS-GPI_Slope[4]	2.678	0.582	1.543	3.813	1.00
WS-GPI_Slope[5]	3.622	0.805	2.052	5.192	1.00
WS-GPI_Slope[6]	4.546	1.141	2.321	6.771	1.00
WS-GPI_Slope[7]	5.461	1.451	2.631	8.291	1.00
WS-GPI_Slope[8]	6.379	1.733	2.999	9.759	1.00
WS-GPI_Slope[9]	7.304	2.003	3.398	11.210	1.00
WS-GPI_Slope[10]	8.237	2.219	3.909	12.564	1.00
$DIC_{marginal}$ = 2144.07					
$DIC_{conditional}$ = 926.40					

Table S5. VCJM results: Using the current value and current slope of the RS-GPI to predict the risk of worsening of disability. Posterior means, 95% credible intervals and Gelman-Rubin's diagnostic (Rhat = potential scale reduction factor), are shown.

	**Est**	**SE**	**2.5%**	**97.5%**	**Rhat**
Longitudinal process: WS-GPI ~ns(time, 3)					
Intercept	1.183	0.074	1.044	1.331	1.00
ns(time, 3)1	-0.200	0.065	-0.325	-0.071	1.00
ns(time, 3)2	-0.248	0.188	-0.625	0.111	1.00
ns(time, 3)3	0.003	0.109	-0.221	0.205	1.00
Error (σ)	0.725	0.011	0.703	0.746	1.00
Survival process: WS-GPI ↔Relapse risk					
Sex(Male)	-1.726	0.608	-2.912	-0.540	1.00
Age at FDE	-0.010	0.013	-0.035	0.015	1.00
Body mass index	0.024	0.018	-0.012	0.059	1.00
Relapse counts	0.164	0.248	-0.319	0.648	1.00
T2 lesion counts	0.885	0.356	0.192	1.579	1.00
RS-GPI_Value[1]	3.863	0.768	2.366	5.361	1.00
RS-GPI_Value[2]	3.087	0.565	1.985	4.189	1.00
RS-GPI_Value[3]	2.284	0.450	1.407	3.162	1.00
RS-GPI_Value[4]	1.447	0.412	0.643	2.250	1.00
RS-GPI_Value[5]	0.592	0.475	-0.335	1.518	1.00
RS-GPI_Value[6]	-0.295	0.635	-1.534	0.943	1.00
RS-GPI_Value[7]	-1.227	0.852	-2.888	0.434	1.00
RS-GPI_Value[8]	-2.185	1.139	-4.407	0.036	1.00
RS-GPI_Value[9]	-3.144	1.471	-6.013	-0.276	1.00
RS-GPI_Value[10]	-4.096	1.734	-7.478	-0.715	1.00
RS-GPI_Slope[1]	6.217	2.124	2.075	10.359	1.00
RS-GPI_Slope[2]	4.410	1.704	1.087	7.733	1.00
RS-GPI_Slope[3]	2.603	1.160	0.341	4.865	1.00
RS-GPI_Slope[4]	0.779	1.019	-1.208	2.766	1.00
RS-GPI_Slope[5]	-1.082	0.859	-2.757	0.593	1.00
RS-GPI_Slope[6]	-2.976	1.313	-5.536	-0.416	1.00
RS-GPI_Slope[7]	-4.882	1.836	-8.462	-1.301	1.00
RS-GPI_Slope[8]	-6.795	2.417	-11.508	-2.081	1.00
RS-GPI_Slope[9]	-8.710	2.959	-14.480	-2.939	1.00
RS-GPI_Slope[10]	-10.628	3.565	-17.580	-3.677	1.00
$DIC_{marginal}$ =5880.66					
$DIC_{conditional}$ = 4904.76					

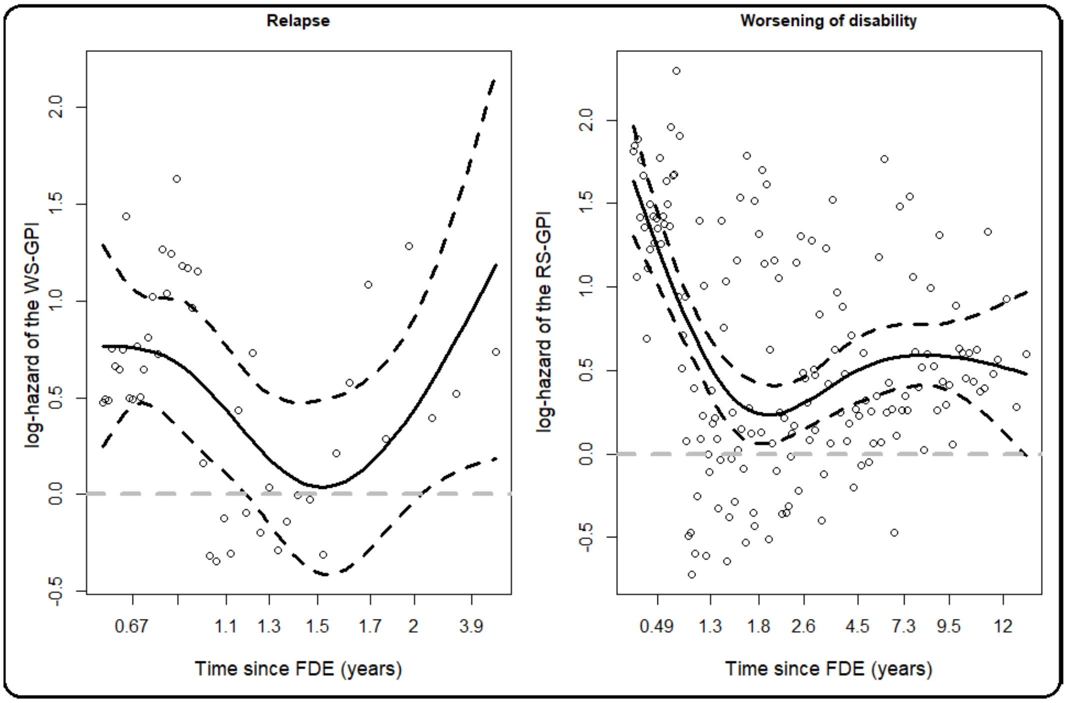

Figure S1. Time-varying coefficients of WS-GPI and RS-GPI, using Schoenfeld residuals. The solid line represents the mean estimate while the dotted lines are the confidence intervals. The dashed grey line represents the coefficient of WS-GPI and RS-GPI where the hazard is constant

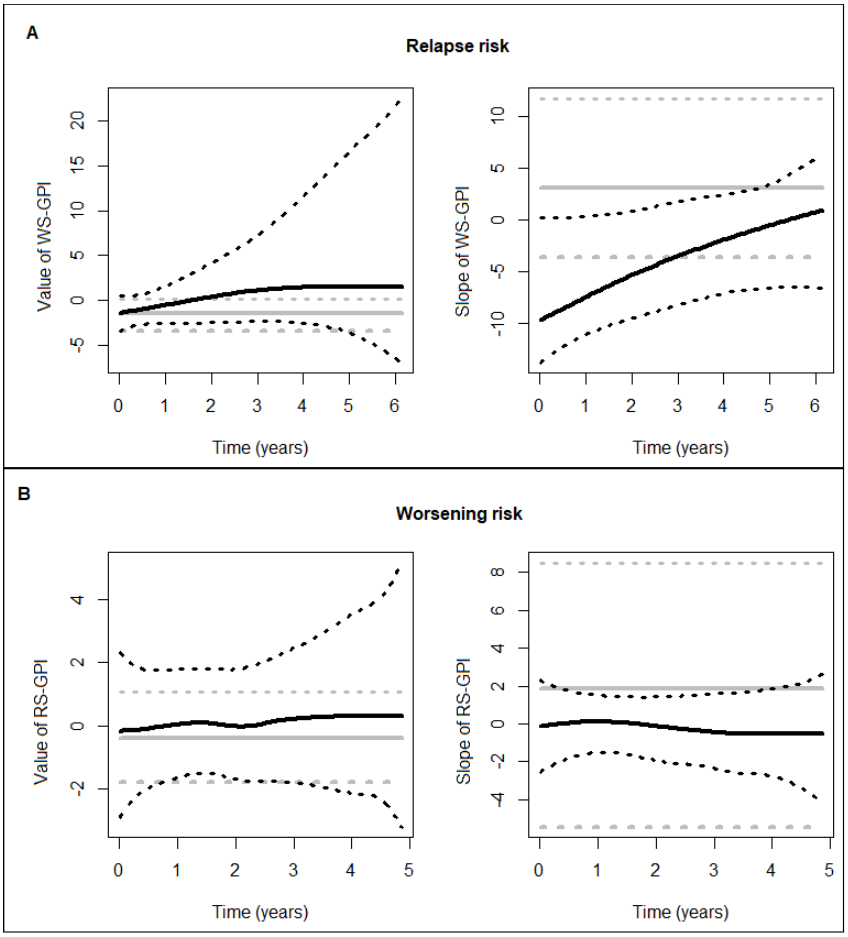

Figure S2. Time-dependent associations using invalid genetic instruments. The residual effects of worsening events on the risk for relapse (A), and the residual effects of relapses on the risk for worsening (B) are shown. These associations were obtained from genetic prognostic indices constructed using top 45 genome-wide SNPs that were not predictive of either endpoint. There is no significant association between relapses and worsening of disability in the presence of invalid SNP-outcome associations. These results do not invalidate our findings from Figure 2.

Appendix C: Model Diagnostic Checks

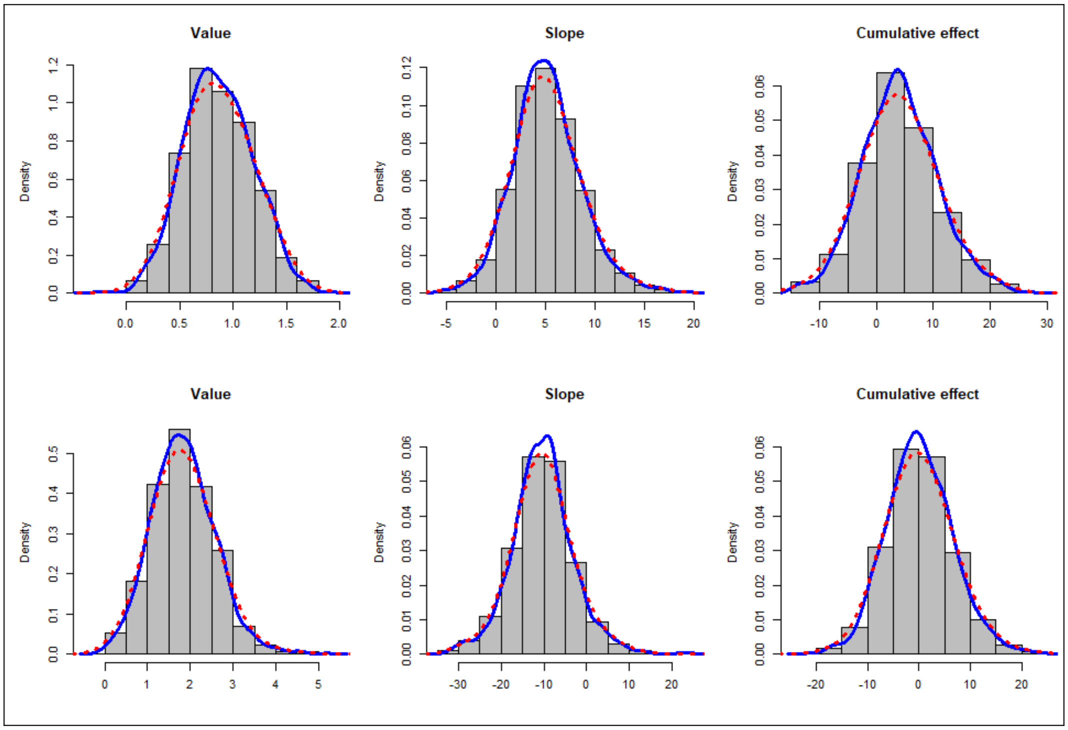

Figure S3. The posterior density of the association parameters in the constant coefficient joint models are normally distributed

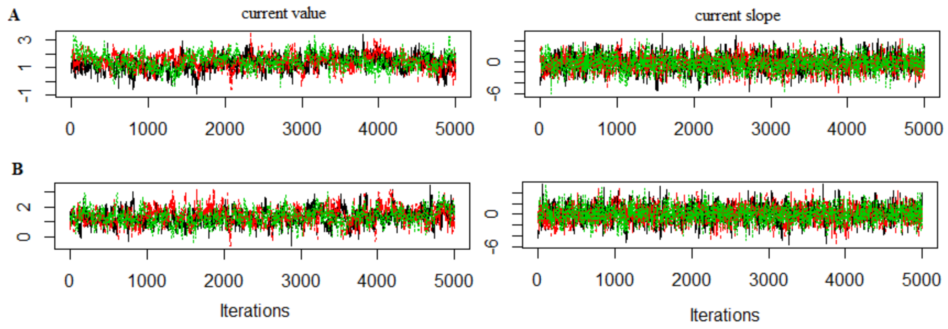

Figure S4. Trace plots of the association parameters indicate better mixing of the Markov chains

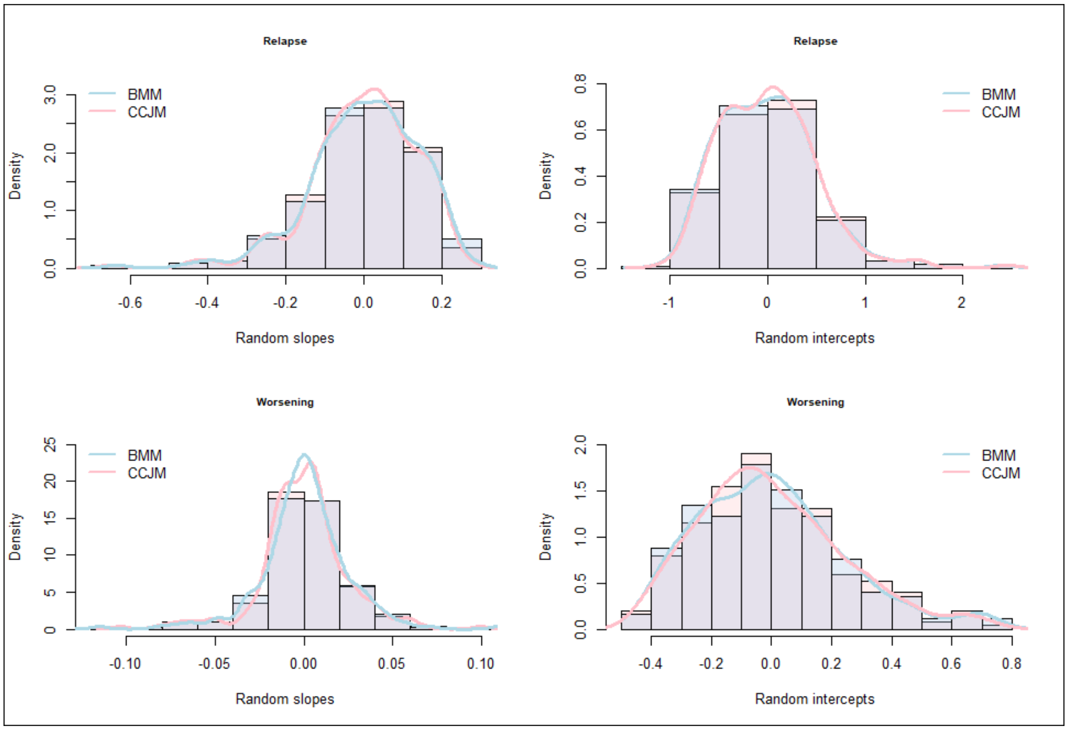

Figure S5. Comparing the distribution of random intercepts and random slopes generated from Bayesian mixed-effects models with those from the constant coefficient joint models.

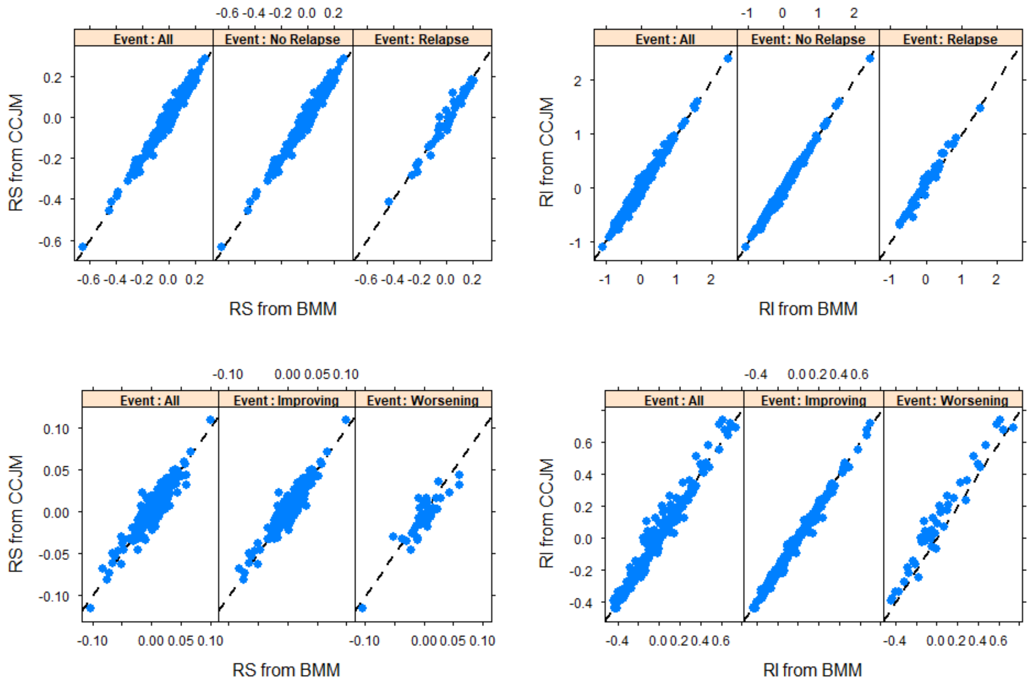

Figure S6. High degree of correlation between random intercepts and random slopes indicate good predictions
